# Evidence of the impacts of pharmaceuticals on aquatic animal behaviour (EIPAAB): a systematic map and open access database

**DOI:** 10.1186/s13750-025-00357-6

**Published:** 2025-03-20

**Authors:** Jake M. Martin, Marcus Michelangeli, Michael G. Bertram, Paul J. Blanchfield, Jack A. Brand, Tomas Brodin, Bryan W. Brooks, Daniel Cerveny, Kate N. Fergusson, Malgorzata Lagisz, Lea M. Lovin, Isaac Y. Ligocki, Shinichi Nakagawa, Shiho Ozeki, Natalia Sandoval-Herrera, Kendall R. Scarlett, Josefin Sundin, Hung Tan, Eli S. J. Thoré, Bob B. M. Wong, Erin S. McCallum

**Affiliations:** 1 Department of Wildlife, Fish and Environmental Studies, Swedish University of Agricultural Sciences, Umeå, Sweden; 2 School of Life and Environmental Sciences, Deakin University, Waurn Ponds, Victoria Australia; 3 School of Biological Sciences, Monash University, Melbourne, Victoria Australia; 4 Department of Zoology, Stockholm University, Stockholm, Sweden; 5 Australian Rivers Institute, Griffith University, Nathan, Queensland Australia; 6 Fisheries and Oceans Canada, Freshwater Institute, Winnipeg, Manitoba Canada; 7 Institute of Zoology, Zoological Society of London, London, UK; 8 Department of Environmental Science, Baylor University, Waco, Texas USA; 9 Faculty of Fisheries and Protection of Waters, University of South Bohemia in Ceske Budejovice, South Bohemian Research Center of Aquaculture and Bioaffiliationersity of Hydrocenoses, Vodnany, Czech Republic; 10 Evolution and Ecology Research Centre, School of Biological, Earth and Environmental Sciences, University of New South Wales, Sydney, New South Wales Australia; 11 Department of Biology, Millersville University, Millersville, Pennsylvania USA; 12 Department of Biological Sciences, University of Alberta, Edmonton, Alberta Canada; 13 Environment Protection Agency, EPA Office of Water, Office of Science and Technology, Washington, USA; 14 Department of Aquatic Resources, Swedish University of Agricultural Sciences, Drottningholm, Sweden; 15 Environment Protection Authority Victoria, EPA Science, Macleod, Victoria Australia; 16 TRANSfarm - Science, Engineering, & Technology Group, KU Leuven, Lovenjoel, Belgium; 17 Laboratory of Adaptive Biodynamics, Research Unit of Environmental and Evolutionary Biology, Institute of Life, Earth and Environment, University of Namur, Namur, Belgium

**Keywords:** Ecotoxicology, Evidence synthesis, Fitness, Medicine, Neurotoxicology, Psychoactive

## Abstract

**Background:**

Over the last decade, pharmaceutical pollution in aquatic ecosystems has emerged as a pressing environmental issue. Recent years have also seen a surge in scientific interest in the use of behavioural endpoints in chemical risk assessment and regulatory activities, underscoring their importance for fitness and survival. In this respect, data on how pharmaceuticals alter the behaviour of aquatic animals appears to have grown rapidly. Despite this, there has been a notable absence of systematic efforts to consolidate and summarise this field of study. To address this, our objectives were twofold: (1) to systematically identify, catalogue, and synthesise primary research articles on the effects of pharmaceuticals on aquatic animal behaviour; and (2) to organise this information into a comprehensive open-access database for scientists, policymakers, and environmental managers.

**Methods:**

We systematically searched two electronic databases (Web of Science and Scopus) and supplemented these with additional article sources. The search string followed a Population–Exposure–Comparison–Outcome framework to capture articles that used an aquatic organism (population) to test the effects of a pharmaceutical (exposure) on behaviour (outcome). Articles were screened in two stages: title and abstract, followed by full-text screening alongside data extraction. Decision trees were designed a priori to appraise eligibility at both stages. Information on study validity was collected but not used as a basis for inclusion. Data synthesis focused on species, compounds, behaviour, and quality themes and was enhanced with additional sources of metadata from online databases (e.g. National Center for Biotechnology Information (NCBI) Taxonomy, PubChem, and IUCN Red List of Threatened Species).

**Review findings:**

We screened 5,988 articles, of which 901 were included in the final database, representing 1,739 unique species-by-compound combinations. The database includes data collected over 48 years (1974–2022), with most articles having an environmental focus (510) and fewer relating to medical and basic research topics (233 and 158, respectively). The database includes 173 species (8 phyla and 21 classes). Ray-finned fishes were by far the most common clade (75% of the evidence base), and most studies focused on freshwater compared to marine species (80.4% *versus* 19.6%). The database includes 426 pharmaceutical compounds; the most common groups were antidepressants (28%), antiepileptics (11%), and anxiolytics (10%). Evidence for the impacts on locomotion and boldness/anxiety behaviours were most commonly assessed. Almost all behaviours were scored in a laboratory setting, with only 0.5% measured under field conditions. Generally, we detected poor reporting and/or compliance with several of our study validity criteria.

**Conclusions:**

Our systematic map revealed a rapid increase in this research area over the past 15 years. We highlight multiple areas now suitable for quantitative synthesis and areas where evidence is lacking. We also highlight some pitfalls in method reporting and practice. More detailed reporting would facilitate the use of behavioural endpoints in aquatic toxicology studies, chemical risk assessment, regulatory management activities, and improve replicability. The EIPAAB database can be used as a tool for closing these knowledge and methodological gaps in the future.

**Supplementary Information:**

The online version contains supplementary material available at 10.1186/s13750-025-00357-6.

## Background

Pharmaceuticals are widely detected in the environment, having been reported in aquatic ecosystems globally [1, 2]. Pharmaceuticals present a particular concern for aquatic animals, with the discharge of human, veterinary, and livestock wastewater effluents being a primary source of contamination. These contaminants can also enter the environment during pharmaceutical manufacturing, through landfill leachates, and run-off from biosolids used in agriculture [2, 3]. Aquatic animals exposed to pharmaceuticals can directly or indirectly bioconcentrate some of these compounds in their tissues [4, 5]. There are now growing calls for the effective management of pharmaceutical pollution in aquatic environments [6, 7]. Yet, for many pharmaceuticals, empirical sublethal ecotoxicological information is lacking, precluding robust ecological risk assessments for aquatic animals [8]. Where ecotoxicity data are available, they are often limited to standard toxicological endpoints (i.e. morphometric endpoints), such as growth, reproductive output, and mortality [8]. It is essential to consider that the effects of pharmaceutical exposure on aquatic animals are likely to be subtle, given that pharmaceuticals are typically detected at low concentrations (low ng/L– low µg/L), are specifically designed to have low-dose effects in their target organisms, and many drug targets are conserved across vertebrate taxa [9]. However, this does not discount adverse environmental impacts, as wildlife may experience unintended, pharmacological (therapeutic-like) or adverse human side effects from pharmaceutical exposure [10–12]. Consequently, a growing body of research is investigating adverse outcomes of pharmaceutical exposure, specifically sub-lethal effects on processes like endocrine signalling, development, bioenergetics, and behaviour [13–16].

In recent years, behaviour has emerged as a key endpoint of interest for researchers and environmental managers assessing the impacts of emerging chemicals of environmental concern, including human pharmaceuticals and veterinary medicines [13, 17, 18]. This is because behaviour is a tractable endpoint, as it is a particularly sensitive indicator for measuring contaminant-induced effects on non-target species, especially when compared to standard ecotoxicological endpoints [19, 20]. Behaviour can also bridge the gap between proximate, sub-organismal, individual-level processes, to ultimate, ecologically relevant, population-level outcomes, which are important for environmental protection goals [16, 21]. However, behaviour is rarely used in a regulatory context [17, 18, 22]. Recent recommendations have highlighted that integrating behavioural endpoints with other adverse outcomes or standard endpoints (e.g. survival, growth) and improving the reliability of behavioural studies will help improve the quality of scientific contributions and utility in regulatory settings [17, 22].

Alongside the increasing use of behavioural endpoints in ecotoxicology, there has been growing awareness that pharmaceuticals specifically designed to modify behaviour are present in the aquatic environment and the tissues of aquatic animals (e.g. antidepressants, anxiolytics, antipsychotics [23–27]). Indeed, many pharmaceuticals are specifically designed to alter behaviour as their primary therapeutic effect (e.g. antidepressants, anxiolytics, antipsychotics), whereas others may inadvertently lead to behavioural changes (e.g. analgesics, hormone therapies) [8, 13]. Widespread environmental contamination with behaviour-modifying drugs, together with increased recognition of behaviour as a sensitive endpoint for ecotoxicology, has culminated in an exponential growth of research focused on the behavioural effects of a multitude of pharmaceuticals on aquatic organisms (e.g [28–32]). For this rapidly expanding field, it is now essential that we synthesise the data being produced and identify focus areas, knowledge gaps, and opportunities for future research.

Here, we have conducted systematic mapping to identify, categorise, and visualise research detailing the effects of pharmaceuticals on the behaviour of aquatic animals. Systematic Evidence Maps (SEMs) help to identify research trends, show knowledge gaps where further primary research is needed, and specify areas with enough data for targeted evidence synthesis approaches (i.e. systematic review, meta-analysis) [33, 34]. Importantly, SEMs have recently been identified as an underutilised tool for chemical risk assessment and decision-making because they can provide a comprehensive summary of literature relevant for future policy while also minimising bias [35]. SEMs are especially valuable for connecting heterogeneous interdisciplinary data, like those used in ecotoxicology and chemical risk assessments, which are beyond the scope, and/or expertise of any one scientist [36]. Therefore, given the rapid expansion of behavioural ecotoxicology and growing interest in behavioural endpoints for chemical risk assessment and management, a SEM is a timely approach for understanding the behavioural effects of pharmaceuticals on aquatic animals.

## Objective of the review

### Primary objective

We aimed to identify and catalogue evidence on the effects of human and veterinary pharmaceuticals on aquatic organism behaviour and present this evidence in an open-access database. The primary review question is, ‘What evidence exists on the effects of human and veterinary pharmaceuticals on aquatic organism behaviour?’ Our SEM has the following elements:

#### Population

Any aquatic animal, that is a metazoan with at least one obligate aquatic phase of its life (e.g. fish, amphibia, aquatic mammal, aquatic invertebrate).

#### Exposure

A human or veterinary pharmaceutical compound.

#### Comparator

A control (i.e. unexposed) or solvent control group of animals.

#### Outcome

A behavioural trait. We define behaviour as organismal kinematic responses, or lack of kinematic responses (e.g. freezing, bursting), to an internal or external stimulus (e.g. foraging in response to hunger [internal] or food [external] stimuli).

### Secondary questions

In addition, our SEM addressed two secondary questions.

1. What knowledge gaps exist that could be future research priorities, and what areas of research have sufficient data for further synthesis?
2. How many articles measure additional endpoints (e.g. sub-organismal, reproduction, growth, survival) alongside behaviour, which could be used to facilitate connections across domains?

## Methods

The reporting of the methodology follows MeRIT to improve author contributions’ granularity and accountability; author contributions will be highlighted in text with their initials [37]. This systematic map is based on the methods described in the previously published protocol [38]. It follows the reporting standards for Systematic Evidence Syntheses in environmental research (ROSES [39]; see Additional File 1). External stakeholders were not engaged in the design of this protocol or the review process. Our SEM has also been pre-registered using the Open Science Framework (OSF) online platform, and the registration is freely available at: 10.17605/OSF.IO/7N92E. This article adheres to the Collaboration for Environmental Evidence (CEE) Standards Guidelines and Standards for Evidence Synthesis in Environmental Management [40].

## Deviations from the protocol

Several deviations from the original published protocol for this systematic map [38] were made. These deviations are summarised as follows:

1. The planned bibliometric analyses and the screening of academic theses were not conducted because of changes to the initial search string during the protocol peer-review process. This resulted in an increase in the total number of search returns and, so too, the total amount of screening effort required for the project. The additional workload meant that this element of the project had to be removed.
2. In the protocol, full-text screening was to be performed in duplicate. This was also changed as a result of the increased number of search returns (i.e. 1,239 articles underwent full-text screening). Instead, 10% of all articles at the full-text screening stage (*n* = 127) underwent duplicate screening to estimate the consistency of eligibility decisions and meta-data extraction of the final EIPAAB database (see ‘Article screening and eligibility criteria’). In addition, every article that was excluded at the full-text screening stage was subsequently cross-screened (i.e. subsequently screened in duplicate).
3. Some questions in the online full-text screening data extraction form (Additional File 2) were removed and/or altered to decrease extraction workload and increase replicability. All changes were made before the full-text screening and data extraction began. These changes did not relate to eligibility criteria; all the changes are detailed in Additional File 3, Table S1.
4. New authors were recruited to the project, and two original authors withdrew from the project (JTO and GCM). The new authors included were: SO, KNF, LML, KRS, ESJT, and NSH.

## Search for articles

### Search terms and strings

ESM and JMM designed the search string with assistance from ML for Web of Science and Scopus to reflect our PECO framework (i.e. Population, Exposure, Control, Outcome elements). The aquatic organism search terms (i.e. population terms) captured broad taxonomic groups for animals that have at least one phase of their life as obligate aquatic (e.g. fish, amphibia, aquatic mammal, aquatic invertebrate), in addition to the common aquatic model species or any species used in Organization for Economic Cooperation and Development (OECD) Toxicity Testing Guidelines (e.g. guppy, medaka, minnow, cladocerans; both common and genus names). Pharmaceutical compound terms (i.e. exposure terms) included general synonyms for medications and specific pharmaceutical classes (e.g. antidepressants, analgesics). Exposure environment terms covered aspects of the experimental environment and the process of exposing animals to a pharmaceutical (e.g. exposure, treatment, tank). Behaviour terms (i.e. outcome terms) included variants of behaviours that could be measured in aquatic animals (e.g. movement, cognition). No search terms were included addressing the comparator (i.e. a control group) as these terms were unlikely to appear in bibliometric records. We instead covered this in our screening process and eligibility criteria. The full search strings used in both Web of Science and Scopus are reported in Additional File 3, Table S2. The search strings were applied to all keywords, titles, and abstracts in both databases. The searches in Web of Science (Core Collection) and Scopus were initially performed on 17 November 2021 (i.e. included anything prior to November 2021) and were subsequently updated on 13 February 2024 to include the rest of articles published 2021 and all of 2022. The terms used in the search string were as follows: (behav* OR personalit* OR courtship* OR “parental care” OR “maternal care” OR “paternal care” OR mating OR “mate choice” OR “mate selection” OR “mate attract*” OR spawn* OR cuckold* OR nest* OR predat* OR antipredat* OR anti-predat* OR escap* OR burrow* OR cryptic OR hiding OR shelter* OR forag* OR feed* OR hunt* OR provision* OR aggress* OR schooli* OR shoal* OR social* OR affiliat* OR defen* OR contest OR dispers* OR migrat* OR swim* OR locomot* OR move* OR “activity level*” OR exploration OR anxiety OR bold* OR scototaxis OR phototaxis OR thigmotaxis OR learn* OR memory OR cognit*) AND (“aquatic animal*” OR “aquatic wildlife” OR “aquatic organism*” OR fish OR fishs OR fishes OR teleost* OR guppy OR guppies OR poecilia OR goby OR gobies OR pomatoschistus OR trout* OR oncorhynchus OR salmo OR minnow* OR pimephales OR cyprin* OR stickleback* OR gasterosteus OR medaka OR oryzias OR danio OR gambusia OR carp* OR cyprinus OR sunfish OR lepomis OR “european sea bass” OR dicentrarchus OR bream* OR pagrus OR silverside OR menidia OR carassius OR herring OR clupea OR cod OR gadus OR killifish OR nothobranchius OR fundulus OR amphibia* OR frog* OR tadpole* OR xenopus OR rana OR turtle* OR chrysemys OR testudine* OR “aquatic insect*” OR invertebrate* OR crustacea* OR mollusc* OR snail* OR mussel* OR bivalv* OR amphipod* OR daphnia OR oyster* OR scallop* “aquatic worm*” OR “marine worm*” OR chronom* OR “marine mammal*” OR “aquatic mammal*” OR zooplankton* OR zebrafish OR mosquitofish OR killifish OR goldfish OR sunfish) AND (“environmental estrogen” OR benzodiazepine* OR SSRI* OR SNRI OR “selective serotonin reuptake” OR “selective serotonin re-uptake” OR “drug residues” OR beta-blocker* OR “beta blocker*” OR anti-anxiety* OR antianxiety* OR psychoactive OR psychiatric OR pharmaceutical* OR medication* OR “prescription drug*” OR “illicit drug*” OR hallucinogen* OR “recreational drug*” OR antidepressant* OR anti-depressant* OR anxiolytic* OR antipsychotic* OR antimanic* OR anti-psychotic* OR anti-manic* OR anti-histamine* OR anti-convulsant* OR anticonvulsant* OR anti-epileptic* OR antiepileptic* OR antihistamine* OR analgesic* OR painkiller* OR “pain killer*” OR “pain relief” OR contracepti* OR stimulant* OR sedative* OR hypnotic* OR narcotic* OR “endocrine disrupting chemical” OR “endocrine disruptive chemical” OR “endocrine-disruptive chemical” OR “endocrine-disrupting chemical” OR “endocrine disruptor” OR edc) AND (expos* OR tank* OR aquari* OR pool* OR treat* OR lab* OR mesocosm* OR dos* OR concentration* OR test*) NOT (“drug discovery” OR “drug development” OR “marine corps” OR fisher* OR “drug design” OR “essential oil”).

### Search filters

No filters for language or document type were used in Web of Science and Scopus. However, only languages with which the co-authors are proficient were included (English, Swedish, Norwegian, Czech, Slovak, Japanese, Polish, Russian). No limit was placed on publication year during the search (except up until 2022), for Web of Science, this resulted in a search range from 1900 to 2022, and for Scopus, a search range from 1834 to 2022.

### Search sources

Our map targeted experimental research articles (i.e. no reviews or meta-analyses). We targeted this type of article because we wanted to build a database of articles where a controlled pharmaceutical exposure has been conducted. We searched for articles in two broad-coverage online databases: Web of Science (Core Collection) and Scopus, which in combination achieved a 95% recovery for benchmark articles (see comprehensiveness estimated below). All searchers were conducted using JMM’s Monash University institution access (for Web of Science, this included the following ‘editions’: SCI-EXPANDED, SSCI, AHCI, CPCI-S, CPCI-SSH, BKCI-S, BKCI-SSH, ESCI, CCR-EXPANDED, and IC).

### Supplementary searches

We supplemented the database searches in two ways: First, we conducted reference searches of key review articles published on the behavioural effects of pharmaceuticals in aquatic animals. For this, JMM and ESM a priori selected six reviews, that focused on the impacts of pharmaceuticals on aquatic organism behaviour (provided in [38]). Second, ESM and the co-author team advertised on social media platforms and mailing lists (e.g. “X” and the Society of Environmental Toxicology and Chemistry Pharmaceuticals Interest Group) that we were seeking articles on this topic (including any well-documented reports from grey literature). Any articles submitted were sent via a simple Google Form to collect basic article information. We did not expect a large grey literature outside of academic or government scientific research sources because aquatic environmental risk assessments conducted for the approval of new pharmaceuticals do not include animal behaviour as an endpoint [8, 17].

### Estimating comprehensiveness of the search

The details of how we estimated search comprehensiveness and sensitivity are detailed in the published protocol [38]. Briefly, we tested the sensitivity using 83 benchmark articles that were expected to be captured by the search string. Our search string recovered 95% of the benchmark articles (i.e. 5% of available data may have been missed).

### De-duplication of results

Search returns from Web of Science, Scopus, and the additional sources were combined, and duplicates were removed in Mendeley Desktop Software (Mendeley Ltd.) before being imported to Rayyan [41], a software designed for article screening. Any remaining duplicates were identified in Rayyan and removed before starting title and abstract screening.

## Article screening and study-eligibility criteria

Articles were included at the title and abstract screening stage based on five eligibility criteria (listed in Table 1). All screeners underwent training at the start of the project, during which eligibility criteria were explained in detail, and several example screenings were performed. Title and abstract screening was performed using Rayyan, and was completed in duplicate by two independent reviewers randomly assigned to each article (12,094 total screenings [including duplicates]; percentage of screenings: JMM 27%, ESM 27%, KNF 12%, JS 12%, JAB 12%, DC 12%, IYL 12%, HT 12%, MM 12%, JTO 12%*, LML 12%, MGB 12%, SO 11%, KRS 11%, GCM 9%*; *left the project after title and abstract screening). Both reviewers had to agree for the article to be included before moving to the full-text screening and data extraction stage. The consistency of the screener decisions was not recorded prior to each deliberation to reach a uniform decision, so a consistency estimate was not made for the title and abstract screening phase. A list of all title and abstract screening decisions and reasons for exclusion are reported in Additional File 4. The full-text screening was completed using Qualtrics Survey Software (Qualtrics, Provo, UT) alongside data extraction. The inclusion decision at the full-text screening stage was based on six eligibility criteria (listed in Table 1). Full-text screening and data extraction were randomly assigned to screeners (1381 total screenings; JMM 10%, ESM 8%, NSH 8%, ESJT 8%, MM 7%, JAB 7%, KNF 7%, LML 7% SO 7%, DC 6%, IYL 6%, KRS 6%, HT 6%, JS 6%, MGB 3%, ML < 1%), as described above, a subset of full-text screening and data-extraction was performed in duplicate (10%, *n* = 127 selected at random). This subset of duplicate screened articles was used for consistency checks to estimate article inclusion decision alignment. For the 127 articles screened in duplicate, there were 18 disagreements, predominantly resulting from issues assessing the compound eligibility (see Additional File 5 for a list of disagreements). In total, 10% of all duplicate-screened articles were excluded incorrectly, while 4% were included incorrectly. As a result of a higher-than-desired false exclusion rate, all articles that had been designated as ‘excluded’ were subsequently cross-screened (by JMM and ESM). After cross-screening, 10% of articles that were initially ‘excluded’, were subsequently changed to ‘include’ (38 of 373). Due to the large number of articles considered in the systematic map, it was not feasible to cross-check all ‘included’ articles at the full-text stage. Thus, we acknowledge a possible 4% false inclusion rate in the project, which would result in approximately 50 articles being incorrectly included in the final database. We highlight that the broader trends and field-related insights gained from the EIPAAB database are likely robust to this small number of false inclusions, but encourage those using the database for targeted research questions, particularly those using a small number of the total studies, to cross-validate the inclusion criteria relevant for their project. Articles that were allocated as ‘discuss’ under the eligibility question (indicating extractor uncertainty) were also cross-screened, and a final inclusion/exclusion decision was made (by JMM). A list of all articles excluded at the full-text screening stage and the reason for exclusion is reported in Additional File 6. For both screening stages, screeners were not assigned articles in which they were listed as authors.

**Table 1:** Eligibility criteria associated question element (i.e. PECO element or other criteria such as language) and the screening stage at which it applies, title and abstract, full text or both

Eligibility criteria	Question element	Screening stage
Uses an aquatic animal. *Animals that have at least one phase of their life as obligate aquatic (e.g. fish*,* amphibia*,* aquatic mammal*,* aquatic invertebrate)*	Population (P)	Both
Uses a wild type animal *An animal that is not genetically modified*	Population (P)	Full text
Uses at least one pharmaceutical compound *A decision tree was used to assist screeners in deciding whether a compound qualifies as a pharmaceutical compound (Figure i*S1)	Exposure (E)	Both
Has a control group *A non-exposed group to which the exposed group is compared and is therefore not a review*,* meta-analysis*,* conference proceeding* etc.	Comparator (C)	Both
Measures behaviour *An organism’s kinematic response*,* or lack of kinematic response (e.g. freezing*,* resting)*,* to an internal or external stimulus (e.g. foraging in response to hunger [internal] or food [external] stimuli)*	Outcome (O)	Both
Is in a language in which our review team is proficient: *English*,* Swedish*,* Norwegian*,* Czech*,* Slovak*,* Japanese*,* Polish*,* Russian*	Language	Both

## Study validity assessment

We collected information on study validity from all included articles during data extraction; however, articles were not excluded from the SEM based on any validity criteria. We collected information on study validity guided by the Criteria for Reporting and Evaluating Ecotoxicity Data (CRED [42]), extracting information directly relating to 10 of the 20 CRED reliability criteria. Specifically, we extracted information relating to Criteria 1 (“Is a guideline method [or modified guideline] used”), Criteria 2 (“Is the test performed under GLP conditions”), Criteria 3 (“[A]re validity criteria fulfilled [control survival, growth]”), Criteria 5 (“Is the test substance identified with name or CAS number…”), Criteria 6 (“Is the purity of the test substance reported…”), Criteria 8 (“Are the organisms well described…”), Criteria 9 (“Are the test organisms from a trustworthy source…”), Criteria 11 (“Is the experimental system appropriate for the test organism…”), Criteria 14 (“Is the exposure duration defined”), Criteria 15 (“Are chemical analyses adequate to verify concentrations of the test substance…”). For a list of which metadata corresponded to each of the CRED criteria and details on why some of the criteria were not considered, see Additional File 3, Table S3 (also detailed in Additional File 7). In addition, we collected the following study validity data not specific to ecotoxicity data: (1) whether animals were randomly assigned to treatment groups, (2) whether behaviour was scored blind to treatment, (3) how behaviour was scored (e.g. manual *versus* automated), (4) if any conflicts of interest were stated. The National Health and Medical Research Council (NHMRC) of Australia list all of these criteria in their 2017 guidelines for “[b]est practice methodology in the use of animals for scientific purposes”. Specifically, in Sect. 3.1, the following conditions are considered flaws in experimental design, “[f]ailure to use randomisation when selecting animals or allocating animals to treatment groups” and “[f]ailure to use blinding when performing an intervention, and when assessing results”. In Sect. 3.4, the “[l]ack of reporting of key methodological parameters that can introduce bias” and “[l]ack of reporting of conflicts of interest that may introduce bias” are also considered flaws.

In total, we had 19 metadata questions relating to study validity (detailed in Additional File 7, in the ‘validity_assessment’ column); we documented aspects of study validity via the CRED reliability guidance and the above additional questions for three reasons. First, behavioural studies in ecotoxicology have been criticised [43, 44] for not following standardised methods or for providing too little data for use in risk assessment procedures. These study validity descriptors will allow us to identify common methodological gaps being overlooked by scientists conducting behaviour-focused studies (e.g. not reporting CAS identifiers, not reporting water quality parameters). Second, scoring behaviour blind to treatment is a standard protocol in behavioural ecology to reduce experimental bias; however, this method may be less prominent for researchers outside of behavioural ecology. Thus, we wanted to identify the number of articles taking this key methodological consideration into account. Third, we included study validity descriptors to improve the utility of the EIPAAB database for future users.

## Data coding strategy

### Data extraction protocol

All articles were assigned a numeric ‘article ID’ that identified the article throughout the title and abstract screening, full-text screening, and the data extraction process. For full-text screening and data extraction, the screening team was assigned a list of articles which contained the article ID, article title, year of publication, journal, and authors (as a CSV file). The screeners used this document to search for and download the articles. The data extraction was coded using an online form (Qualtrics Survey Software; designed by ESM and JMM with input from all co-authors). Before the allocation of full-text articles, all screeners were first trained using a pilot screening with 10 randomly selected articles. This was done to clarify uncertainty for extractors, and to test the efficacy and functionality of the full-text screening and data collection form (as reported in [38]). Where metadata/extraction data were missing or unclear, it was coded as “Not reported/not specified/not stated/not disclosed”; in addition, for some questions, extractors were given the option to specify “Other”, a free text option to leave comments which were checked by JMM and ESM, as well as a more general ‘Elaboration and comments section’ (Q62) at the end of the online full-text screening and extraction form for which extractors could leave questions (see Additional File 2 for a list of all extraction questions and options). The authors of the articles were not contacted to recover missing information. The article metadata were extracted in the following survey sections (full survey structure supplied in Additional File 2):

1. Details about the screener and article: information on the screener and the article being extracted (e.g. screener initials, article ID, DOI).
2. Inclusion criteria: data on the inclusion criteria (see Table 1). If the reviewer chooses to exclude the article, they skip the remaining data extraction.
3. Study species: data on the aquatic organism(s) studied (e.g. species name, animal source, sex, life stage).
4. Pharmaceutical compound(s): data on the pharmaceutical compound(s) being studied and the exposure environment (e.g. compound name, route of exposure, dosage, exposure duration).
5. Behavioural endpoints: data on which behaviours were measured. Behaviours are first categorised into 10 broad categories (e.g. movement/activity, aggression, foraging, boldness; see Table S4 for full list) and then into more specific subcategories (2–12 per parent category; 62 total), to extract more detail on how the behaviour was measured (e.g. within movement/activity: normal locomotor activity, abnormal movements, dispersal/migration; see Additional File 3, Table S4 for full list and definitions).
6. Connecting across biological scales: data on whether the article also measured any sub-organismal traits (e.g. hormone concentrations, mRNA transcription) and/or endpoints capturing growth, reproduction, or survival. We included these questions to increase the utility of the EIPAAB database.
7. Validity: data describing the study validity (see ‘Study validity assessment’ for further details).
8. Research motivation: the primary scientific motivation of the article was allocated to environmental (i.e. focus on predicting/measuring the effects of environmental pollution on wildlife; ecotoxicology), medical (focus on improving human or veterinary medical practice), or basic research (focus on understanding biological phenomena or methodological development with no overt applicational claims for medical or ecotoxicological purposes).

### Data processing

The data collected by the online survey form were downloaded as CSV files and imported into R (version 4.2.3, in the R studio environment, Build 463; [45]) for data processing (by JMM). Errors with DOI and ‘article ID’ (i.e. unique project allocated IDs) were identified by cross-referencing titles, DOIs, and article IDs with the article allocation list given to extractors. The database was then re-shaped to a long format, where each article was given a row for each tested chemical and each tested species, in other words, a row for each unique species-by-compound combination. Compound names and species names were then assessed for possible synonyms or typographical errors. For compounds, this was done by searching compound names in the PubChem database [46], and collating PubChem CID, PubChem name, CAS, and synonyms (Python script by JMM is provided on Github; https://github.com/JakeMartinResearch). These identifier metadata were then used to evaluate possible synonyms or typographical errors in the database (e.g. different compound names that shared a CAS number). For species, this was done using the National Centre for Biotechnology Information (NCBI) Taxonomy database [47]), with each species name searched, and the taxonomy ID, current taxonomic name, and full lineage collated; these species metadata were used to evaluate possible synonyms or typographical errors in the database. For articles that had multiple species, the compound and behaviour data were cross-checked to make sure that the answers given by extractors applied to all species, if they did not, they were adjusted. This was necessary as the survey form did not allow extractors to give separate answers for different species within the same article. All survey questions with an ‘Other’ option to provide a free-text based alternate response (e.g. study motivation, behavioural classification, methods used to score behaviour; see survey form linked as Additional File 2) were then assessed by JMM and, where appropriate, were re-assigned to existing categories or were grouped into new categories (see Additional File 3, Table S4–5 list of new categories).

### Consistency estimates

In total, there were 84 duplicate screened articles included, which represented 305 rows of data (i.e. each unique species-by-compound combination). To estimate the consistency of metadata extraction, JMM calculated the alignment between each survey question within each unique species-by-compound combination. When the answer from extractors matched exactly, the data were assigned a ‘1’, if it did not match they were assigned a ‘0’. The median consistency across all metadata was 94.8% ± 8.8%, ranging from 60.8 to 100% (a list of consistency for all metadata is reported in Additional File 3, Table S6). Data that were implicitly consistent (e.g. article ID, DOI, species name, compound name) or not consistent (e.g. screener name), were not included in estimates of the median consistency. As a result of some of the specific behavioural classifications having low consistency (median 95.8%, range 67.6–99.3%; see Additional File 3, Table S6), a Boolean value (1 or 0) for categorisation only at the broadest level of the behavioural class was created, which had higher consistency (median 98.6%, range 75.6–99.3%; see Additional File 3, Table S6). The reason for low consistency for some of the metadata extraction is discussed below in the limitations section. We have opted to maintain all metadata in the database regardless of estimated extraction consistency, but we suggest that those using the EIPAAB database check the level of consistency for the metadata they plan to use, and decide whether it is appropriate for their individual usage.

### Additional metadata to increase usability

To aid in cross-article comparison and to increase the usability of the database, the following additional information was added to the EIPAAB database:

1. Standardised concentrations were added to the database, which converted the original concentration units reported by the authors to one of six standardised units (original units and values were also maintained). Specifically, the following conversions were made: mass/volume measures to µg/L, volume/volume measures to µL/L, mass/mass measures to µg/g, mole units to µM, molarity (mole/volume) units to µM/L, and dimensionless units of concentration to ppm.
2. Compounds were assigned to a therapeutic classification system, specifically the Anatomical Therapeutic Chemical (ATC) classification tree (hereafter ATC; [48]). The ATC classifies active ingredients of drugs according to the organ or system on which they act and their therapeutic, pharmacological, and chemical properties. The ATC classification was selected as it is widely used, covers many compounds in the EIPAAB database (305 of 426 compounds), and has a simple classification structure. For compounds that returned multiple ATC classification trees, the trees were collated. ATCs were pulled from PubChem by JMM, by searching each compound name, extracting the resulting PubChem substance ID (up to 150), and searching classification information for each SID (Python scripts by JMM are provided on Github; https://github.com/JakeMartinResearch). In addition to the full classification tree (as a semicolon-separated list), the classifications are also provided at each level of the tree separately (e.g. 5 ATC classification levels) to make the data more accessible (see Additional File 7 for details).
3. Additional species metadata were added to the EIPAAB database from the International Union for Conservation of Nature’s (IUCN) Red List of Threatened Species [49]. Specifically, JMM and MRM searched each species name in the IUCN Red list, and for those with an associated IUCN Red List report, the IUCN report DOI, IUCN Status, IUCN report publication year, geographic range, population trend, habitat type, and movement patterns were collated (see Additional File 7 for details of each data type).
4. Additional bibliometric metadata from Web of Science and Scopus were collected by JMM (05/07/2024), using a search of the full DOIs list across both online databases (*n* = 894), or by searching the title if the article did not have a DOI (*n* = 7). A total of 879 articles were located on Web of Science (Core Collection), and the extracted metadata included: journal abbreviation (ISO), author keywords, unique Web of Science ID, Web of Science Categories, Web of Science Research Areas, number of cited references, and number of times the article was cited (across all databases). A total of 888 articles were located on Scopus, and the extracted metadata included: journal abbreviation, author keywords, Scopus EID, and number of times the article was cited.

## Data mapping method

We summarise the available research at three levels: (1) the article level, represented as ‘article_id’ in the database; (2) the population level, represented as ‘unique_population_id’ (i.e. article id + species name); and (3) the species-by-compound level, represented as ‘unique_row_id’ in the EIPAAB database (article id + species name + compound name). The level at which our summaries were made depended on the level at which those metadata were extracted and/or applied to the article. For example, metadata like the publication year, conflict statements, and water quality were extracted and summarised at the article level (*n* = 901). Metadata like species life stage, sex, and source were extracted and summarised at the population level (i.e. unique_population_id; *n* = 935), because a single article can have multiple species. Metadata like exposure duration, exposure concentration, and category of behaviours measured were extracted and summarised at the species-by-compound level (i.e. unique_row_id; *n* = 1,739) because in cases where multiple species were used, different exposures and behaviours can be, and were, assessed. The level at which metadata were extracted is listed within Additional File 7, and how this was applied to summarise the data is illustrated in Additional File 8 (i.e. R script). We also performed many of our summaries with respect to the motivation for the study. During metadata extraction, we categorised each article based on its primary motivation, as either environmental (i.e. focus on predicting/measuring the effects of environmental pollution on wildlife; ecotoxicology), medical (focus on improving human or veterinary medical practice), or basic research (focus on understanding biological phenomena or methodological development with no overt applicational claims for medical or ecotoxicological purposes). We did so because we predicted the motivation of the research to strongly influence many aspects of the study design, such that some of our summary data would be insightful only if applied within a given study motivation. For example, we would expect the applied doses to be very different in an environmentally motivated study compared to a medically motivated study. Knowledge gaps (i.e. unrepresented or underrepresented subtopics that warrant further primary research) and knowledge clusters (i.e. well-represented subtopics that are amenable to full synthesis via systematic review) were identified by comparing the relative number of articles/exposures within the database that focuses on a given species/compounds/behaviour to identifying any with topics with low or relatively high occurrence, respectively. All data summary methods are explained in detail in Additional File 8, which is also designed to act as a starting point for anyone who wishes to use the EIPAAB database for their own projects.

## Results

### Overview of the evidence base and temporal trends

**Fig. 1:**
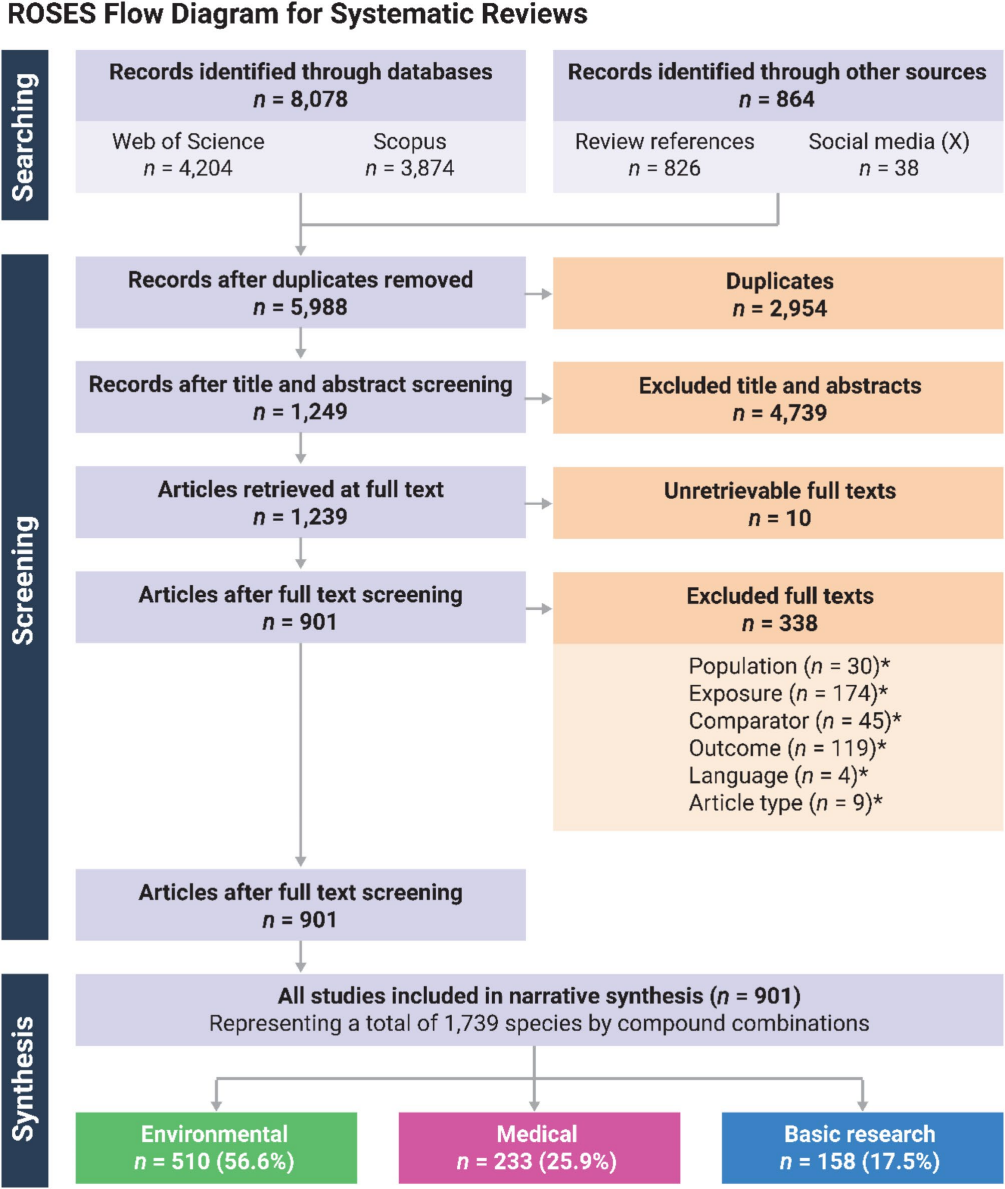
Flow diagram for the SMAP and EIPAAB database, showing the article numbers at each step of the process (i.e. searching, screening, and synthesis). This figure is based on the Reporting Standards for Systematic Evidence Syntheses (ROSES) flow diagram for systematic reviews, version 1.0 [50]. This is also available as a single PDF (Additional File 9). *The total number of articles for each full-text exclusion criterion includes multiple reasons allocated to a single article; we also expected that when articles failed to meet multiple exclusion criteria,* screeners may not have indicated every reason for exclusion (e.g. if the article was the wrong article type)*

In total, 901 articles—representing 1,739 unique species-by-compound combinations—were included in the final EIPAAB database. After collating articles from all sources and de-duplication, we screened a total of 5,988 unique articles for possible inclusion in the systematic map and database (Fig. 1). In brief, 4,739 articles were excluded after title and abstract screening, 338 articles were excluded during full-text screening and data extraction, and 10 articles were unretrievable for full-text screening (overall inclusion rate of 21%; Fig. 1). Most articles were excluded at the full-text screening stage for not having a compound of interest (i.e. exposure: *n* = 174; Fig. 1) or for not measuring a behaviour (i.e. outcome: *n* = 119; Fig. 1).

**Fig. 2:**
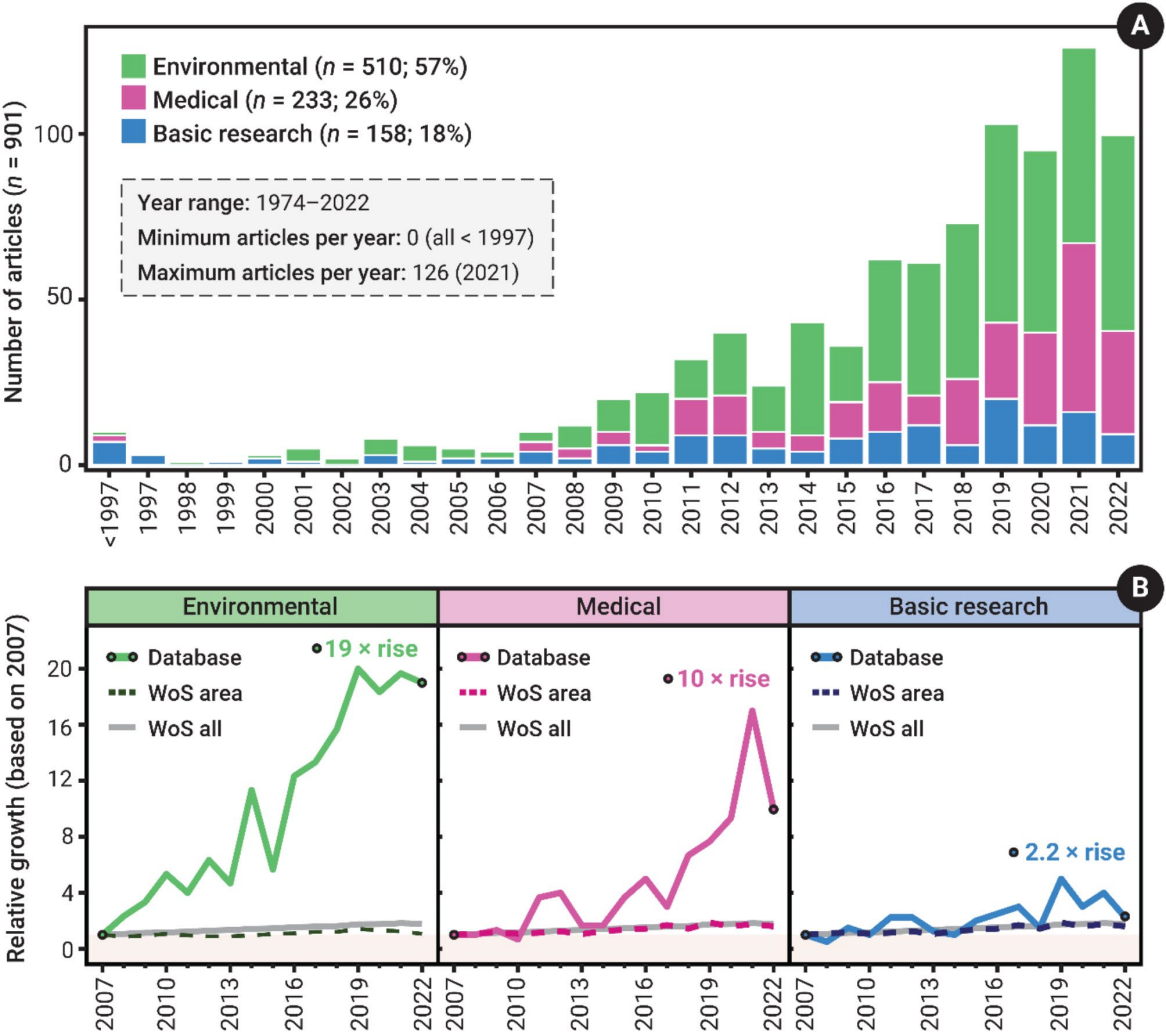
**(A)** The total number of articles included in the EIPAAB database by publication year (articles published before 1997 were grouped; total range 1972–2022). Study motivation is represented by the stacked colours within the bar chart (Environmental = green, Medical = pink, Basic research = blue, stacked in that order). **(B)** The relative growth in the number of articles per year from 2007–2022 based on 2007, as compared to the respective Web of Science Research area (Web of Science area: ‘Environmental Sciences & Ecology’ or ‘Neurosciences & Neurology’), and Web of Science global publication trends (Web of Science all), for Environmental, Medial and Basic research articles in the database

Regarding study motivation, 510 articles had an environmental motivation (56.6%), 233 had a medical motivation (25.9%), and 158 had a basic research motivation (17.5%). The included articles date from 1974 to 2022, with a steep rise in the number of articles around 2007 (Fig. 2A). To specifically assess the growth of research on pharmaceutical impacts on animal behaviour, we compared the relative increase in articles over the last 15 years in the systematic map (2007–2022), against that of the most common Web of Science Research Area, as well as all researcher areas in the Web of Science Core Collection (i.e. an overall publication trend). This was done for each study motivation separately (see Additional File 8 for full details and Additional File 10 for the search results). For articles allocated to the environmental study motivation, the most common Web of Science Research area was ‘Environmental Sciences & Ecology’ (65% fall within this research area); for those allocated to medical and basic research, it was ‘Neurosciences & Neurology’ (47% and 39% fall within this research area, respectively). The growth rate of research articles addressing the impacts of pharmaceutical impacts on animal behaviour with an environmental focus far outpaces that of the broader research area of ‘Environmental Sciences & Ecology’ and the overall publication trend from 2007 to 2022 (Fig. 2B). The growth in research with a medical focus also outpaced the broader research area of ‘Neurosciences & Neurology’ and overall publication trends, but this was only evident from 2018 to 2022 (Fig. 2B). The growth in research with a basic research focus did not consistently deviate from the broader research area of ‘Neurosciences & Neurology’ or overall publication trends (Fig. 2B).

## Mapping characteristics of the population, exposure, and outcomes

### Study species (population)

**Fig. 3:**
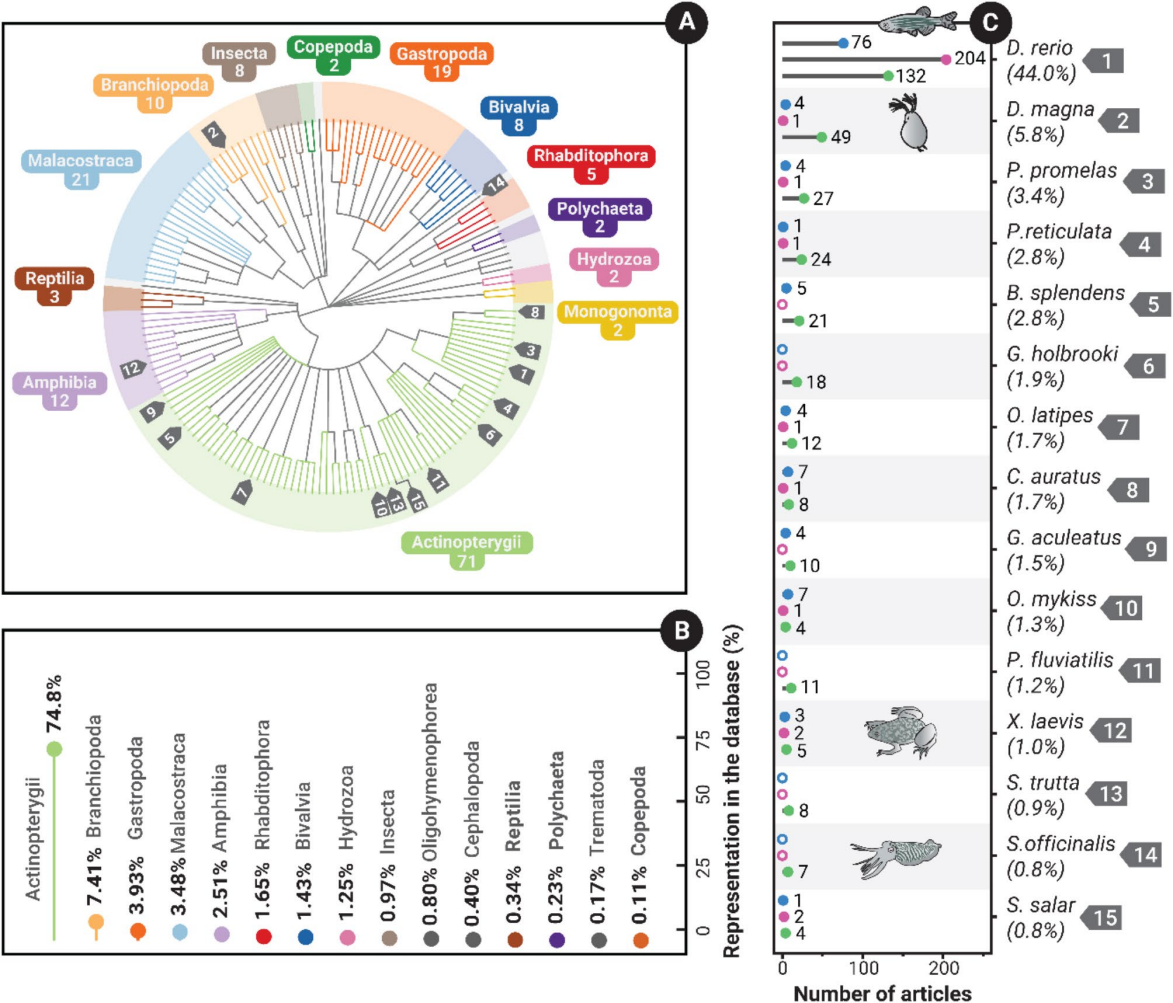
**(A)** Cladogram showing all species included in the EIPAAB database. All classes with more than one species are shown in distinct colours (those with a single species are light grey). The numbered labels 1–15 represent each of the top 15 species represented in panel **C**. **(B)** The 15 most common taxonomic classes in the evidence database. The colours are unique to each phylum and apply across both plots A and B. **(C)**. The 15 most common species used in articles within the evidence database. The percentage value given under the species name is the percentage of total articles, and the counts within the plot are the number of articles for each species by study motivation (Basic research = blue, Medical = pink, Environmental = green, in that order). The open circles are cases of zero articles. The accompanying species images indicate the first occurrence of a distinct taxonomic class in the top 15 species (i.e. Actinopterygii [1st ], Branchiopoda [2nd ], Amphibia [12th ], and Cephalopoda [14th ])

Collectively, the database includes 173 different species from 21 classes (Fig. 3A). In terms of taxonomic diversity, 41.0% of the species present in the evidence database belonged to the clades *Actinopterygii* (i.e. ray-finned fishes), 12.1% to *Malacostraca* (i.e. soft-shelled crustaceans), 11.0% to *Gastropoda* (i.e. gastropods), 6.9% to *Amphibia* (i.e. amphibians), and 5.8% to *Branchiopoda* (e.g. fairy shrimp, water fleas)—all other clades represent less than 5% of the total distinct species (Fig. 3A). Regarding the representation in the evidence base (i.e. how often they were studied), *Actinopterygii* was by far the most common, representing 75.4% of all data in the database; all other clades represented less than 10% of the data included in the database (Fig. 3B). The most common species in the database was the zebrafish, *Danio rerio*, being included in 44.1% of all articles, which is almost a factor of 10 higher than the next most common species, *Daphnia magna* (5.8%; the top 15 most common species shown in Fig. 3C). Interestingly, many species were only used in a single article (103/173), with very few being used in more than 5 articles (17 species; Fig S2).

Taxonomic usage and representation also differed by study motivation; compared to medical articles, those with an environmental and basic research motivation showed a more even spread of taxa, although all had a very strong skew towards ray-finned fishes (Fig S3; Fig. 3C). Considering the total number of articles identified per study motivation, environmental and basic research included substantially more species than medical research (Environmental = 143:510; Medical = 26:233, Basic research *=* 43:158, species: articles).

There was an overrepresentation for species from freshwater habitats compared to marine (80.4% *versus* 19.6%), although this was less obvious in environmental and basic research (Table S7). There was also an overrepresentation for studying animals at the adult life stage (53.3%), compared to juveniles (14.8%), larvae (26.4%) and embryos/eggs (5.5%). This was broadly consistent across all study motivations, although environmental articles had a more balanced representation of life stages (Table S7). The use of female and male animals, when reported, was roughly equal (44.9% *versus* 55.1%), and this was constant across all study motivations (Table S7). Overall, the most common source of study animal was commercial suppliers/fish farms (38.0%), followed by a lab stock with undisclosed origin (26.6%), collection from the wild (24.4%), lab stock from a commercial supplier (6.9%), and lab stock from a wild population (4.1%). The animal source did, however, vary by study motivation, with environmental articles having the highest representation of wild-collected animals and less sourced from commercial suppliers or fish farms (Additional File 3, Table S7).

Importantly, sex, life stage, or animal source were not obtained from all articles. In some cases, these data were not reported at all, or were not reported in sufficient detail to extract and add to the database (see Table 2 for details). The reporting of species-related metadata was considered an aspect of study validity/quality and is discussed in more detail below. With that said, the number of species with missing metadata is also important in interpreting the overall population trends, so this information has been included in the summary table (Additional File 3, Table S7). IUCN data was also not available for all species (106 of 173 had IUCN data), which should be considered when interpreting species IUCN red list metadata and habitat data.

Pharmaceutical compounds and exposure (exposure).

**Fig. 4:**
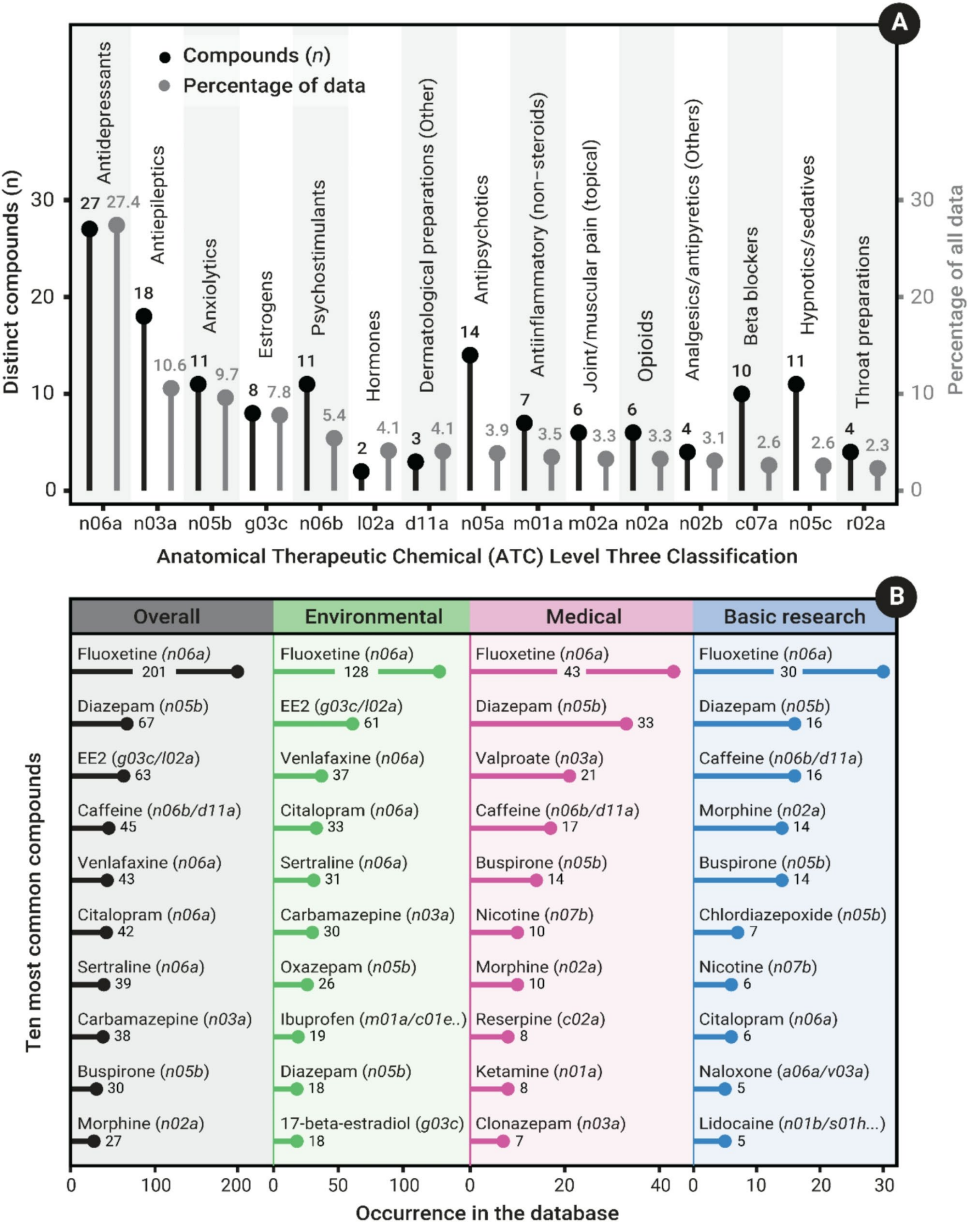
**(A)** The 15 most common level three ATC pharmacological groups, as shown by the number of distinct compounds within each group (black), and overall percentage of occurrence in the EIPAAB database (grey). The x-axis lists the group’s ATC code, while a simplified version of the ATC name is given inside the plot. Note that the total percentage may exceed 100, as each compound may have multiple classifications. **(B)** The 10 most common compounds in the database overall and for each study motivation (Environmental, Medical, and Basic Research), the code in brackets following the compound name are the level three ATC pharmacological groups associated with the compound

Overall, 426 different pharmaceutical compounds were included in the evidence database. The majority of articles used a single compound (*n* = 624, 69.3%), and very few used more than 5 (*n* = 38, 3.9%), with a similar trend in the number of compounds used across study motivations (Additional File 3, Fig S4). We present the compound data in two ways, in terms of the diversity of compounds (irrespective of the number of articles studying them in the EIPAAB database), and their percentage overall representation in the EIPAAB database. In terms of compound diversity—using the WHO Anatomical Therapeutic Chemical (ATC) classification tree—the database includes compounds from all pharmacological groups at the broadest ATC level (14 groups). At this ATC level (i.e. 1st ATC level), the pharmacological group with the most compounds was ‘nervous system’, with 43% of all classified compounds belonging to this group, followed by ‘cardiovascular system’ and ‘alimentary tract and metabolism’ (Fig S5). At the 3rd ATC classification level, antidepressants, antiepileptics, and antipsychotics have the highest number of compounds, at 27, 18, and 11 distinct compounds, respectively (Fig. 4A). In terms of overall percentage representation in the EIPAAB database, compounds within the ATC level one group ‘nervous system’ made up 71.9% of all data, followed by ‘genito urinary system and sex hormones’ (13.5%) and ‘cardiovascular system’ (10.6%). At the 3rd ATC level, antidepressants (27.4%), antiepileptics (10.6%), and anxiolytics (9.7%) were the most common (Fig. 4A). Overall, the most common compound was fluoxetine (antidepressants), which made up 11.5% of all data in the EIPAAB database (see Fig. 4B for the top 10 most common compounds). There were obvious differences in compound use based on study motivation (Fig. 4B). For example, 17 − alpha − ethinylestradiol (EE2) was the third most common compound overall (63 occurrences), but this was almost entirely driven by environmental research (61 occurrences; Fig. 4B). Medical and basic research shared a more similar preference for compounds than they did for environmental research (Fig. 4B). It is important to highlight that not all articles had an assigned ATC classification (307 of 428 had an ATC classification; 72%); thus, all summaries based on ATC do not include all available compounds within the database.

**Fig. 5:**
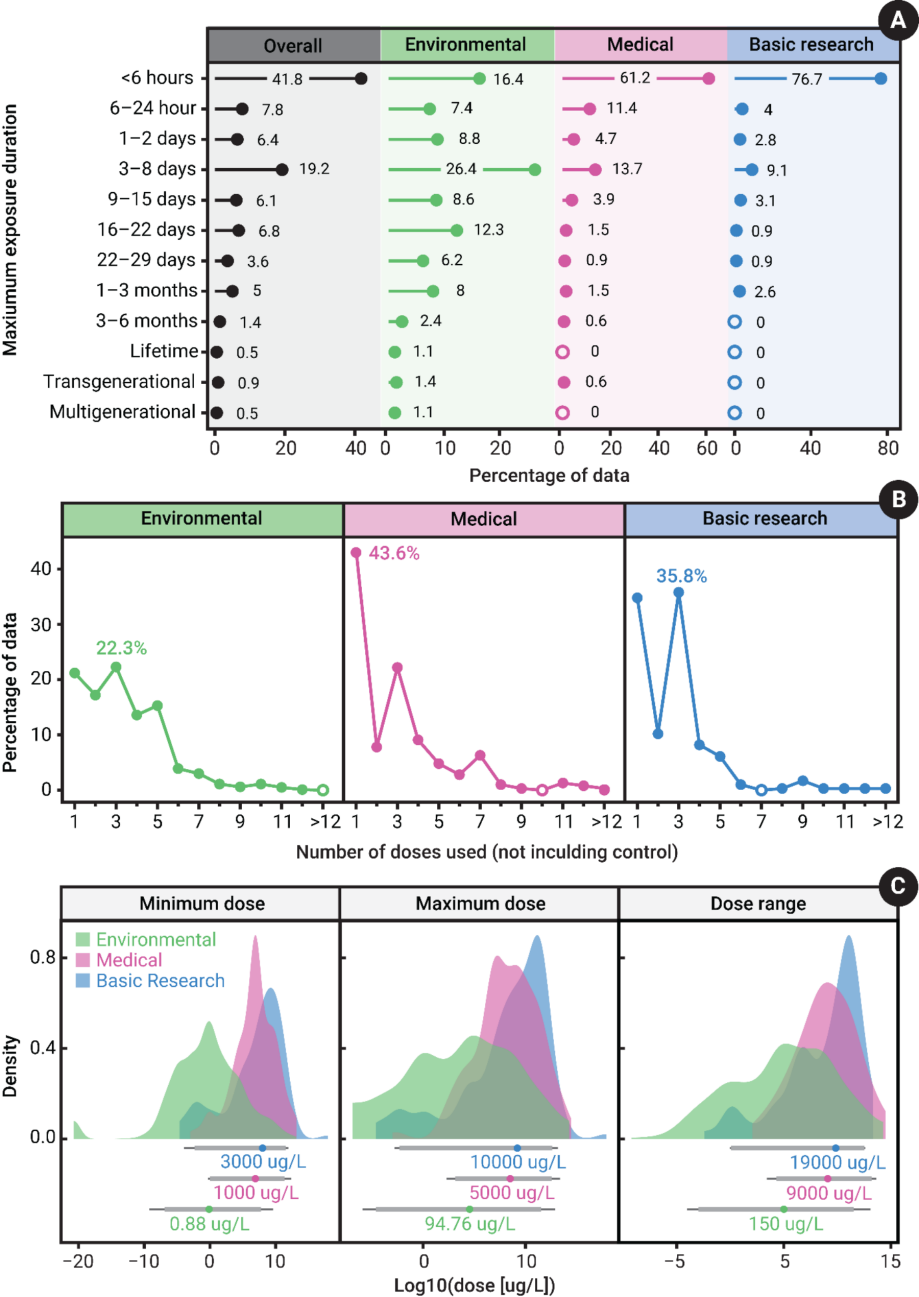
**(A)** The duration of exposures used by articles in the database. The plot is split by the overall percentage breakdown and those for each study motivation. The percentage values are calculated within each study motivation. **(B)** The number of different doses used (excluding the control), as shown by study motivation. The percentage values are calculated within each study motivation. **(C)** The distribution of minimum and maximum dose used, as well as the within-study dose range (i.e. maximum– minimum). The x-axis (dose µg/L) is plotted on a log10 scale for the density plots and ‘eye plots’. The eye plot shows the median, 89, and 95% intervals. The text with the eye plot shows the raw (untransformed) median value and is used to aid in comparisons across study motivations

**Fig. 6:**
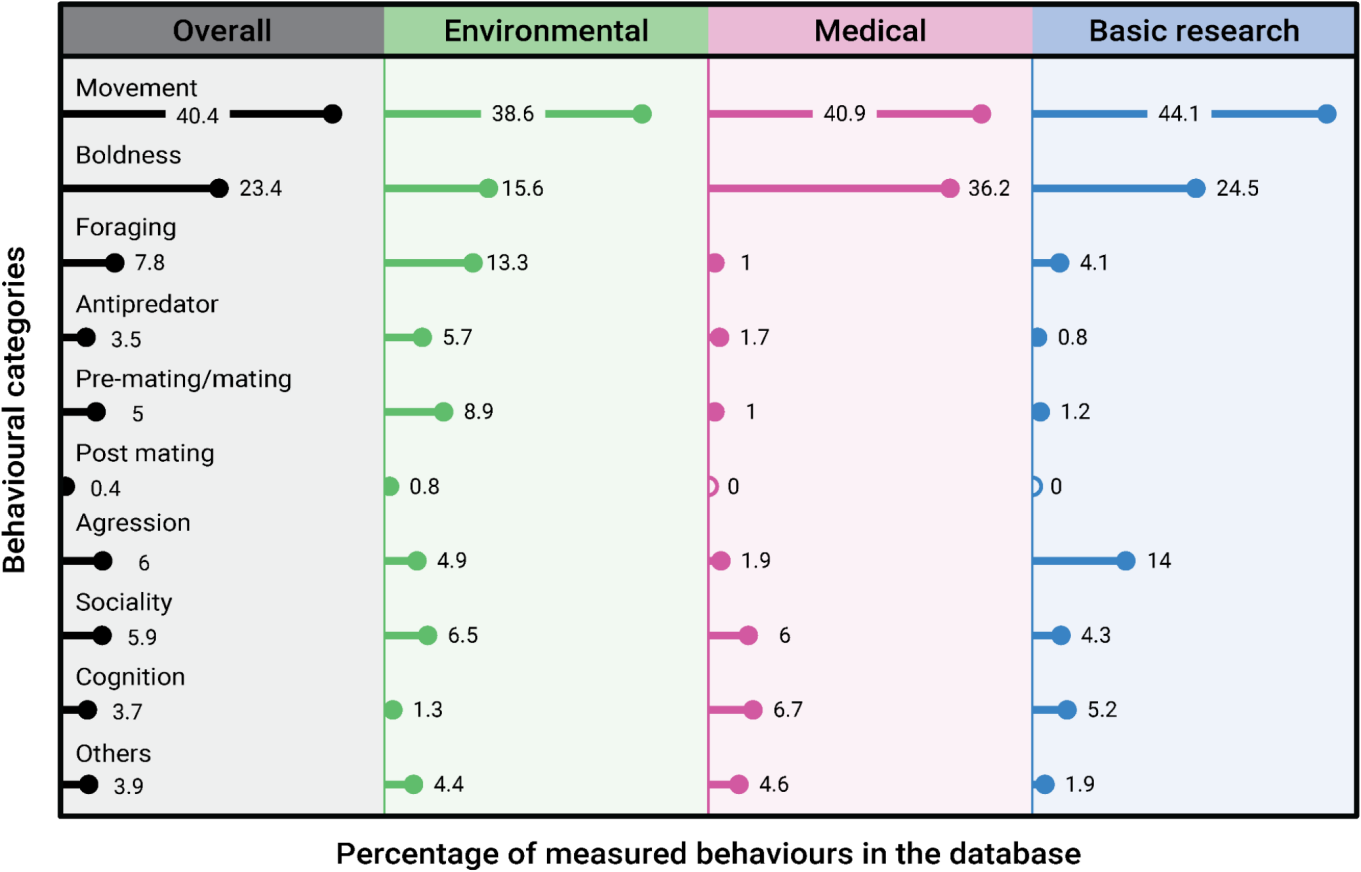
The percentage measurement of different behavioural categories. The plot is split into the overall percentage breakdown and those for each study motivation. For a list of all sub-categories of behaviours and definitions, see Additional File 3, Table S4 and Fig S6

Overall, 22.6% of articles included mixture exposures in addition to single compound exposure. The use of mixture exposures differed substantially by study motivation. Specifically, medical articles had a much higher rate of mixture exposure (48.4%) compared to basic (25.4%) and particularly environmental research (12.8%). This is likely a result of medical-based articles investigating potential treatments for various psychological/neurological conditions (e.g. epilepsy), in which a phenotype for the psychological/neurological condition of interest is induced using a compound exposure and another compound is subsequently administered to alleviate the phenotype. Most exposures were solely waterborne (86.8%), as compared to other exposure routes (e.g. injection, dietary; 12.7%) or a combined exposure with multiple routes (0.9%). Exposure duration was most often acute (i.e. < 96 h), with very few studies using exposure durations over a month (only 8.3%; Fig. 6A). However, there were notable differences between the study motivations. Medical and basic research articles typically employed exposures less than 6 h (61.2 and 76.7%, respectively), and almost never over 3 months (0.6% and 0%, respectively; Fig. 5A). On the other hand, environmental articles had more variation in the maximum exposure durations, with the most common being between 3 and 8 days (26.4%) and more examples of exposures exceeding 3 months (6%; Fig. 5A). Further, overall, most studies exposed animals to a single dose of the compound (29.7%), and very few used more than 5 doses (only 9.8%; Fig. 5B). For environmental research, there was a more even spread in the percentage of articles that included up to 5 doses (15.3–22.3%; Fig. 5B). Broadly speaking, the concentrations used varied substantially, both within and across study motivation (Fig. 5C). Generally, environmental studies used much lower concentrations (both the minimum and maximum dose) and had a smaller within-study dose range (Fig. 5C). Basic research studies used the highest concentrations and had the highest within-study dose range (Fig. 5C). With that said, there was still substantial overlap in the concentrations used between study motivations, which could help facilitate across-discipline comparisons (although this should be checked explicitly at the compound level). Almost all exposures were conducted in indoor laboratory settings (99.4%) *versus* in a semi-controlled outdoor environment (0.3%) or in the wild (0.2%).

### Behavioural endpoints (outcome)

We classified behaviour into 10 overarching categories and 62 sub-categories (2–12 sub-categories within each parent category; a full list of sub-categories and descriptions is given in Additional File 3, Table S4). The 10 over-arching categories were: (1) movement and locomotion, (2) anxiety and boldness, (3) foraging/feeding, (4) antipredator behaviour, (5) pre-mating and mating behaviour, (6) post-mating behaviour, (7) aggression, (8) sociality, (9) cognition/learning, and (10) other behaviours not categorised (see Additional File 3, Table S4 for list). Typically, only one of these behavioural categories was assessed following exposure (69.3%), with few cases assessing more than 3 behavioural categories after exposure (7.8%); this trend was seen within all study motivations. Overall, movement and locomotion behaviours were the most common responses measured (40.4% of all recorded behaviours), followed by boldness and anxiety-related behaviours (23.4%); all other overarching behavioural categories each represented less than 10% of the data. The preference for movement/locomotion and boldness/anxiety-related behaviours was present in all study motivations, the preference for testing the other 7 categories was more variable (Fig. 6). Environmental research had a more even spread of research across the 10 behavioural categories (Fig. 6). Overall, the behavioural groups that have seen the least research attention are post-mating behaviours (e.g. parental care; <1%), antipredator behaviours (3.5%), and cognition and learning (3.7%). Within this manuscript, we will not detail the specific breakdown of each behaviour sub-category, but this information is provided for each study motivation in Additional File 3, Fig S6.

Almost all behaviours were assessed in a laboratory setting (99%), with less than 1% of measured behaviour being conducted in an outdoor natural setting (in an open natural setting or restricted natural setting). This almost complete preference for studies in laboratory settings was present regardless of study motivation (98.7%, 99.6%, 99.7%, environmental, medical, and basic research, respectively). Overall, only 22% of behavioural measures were conducted within a social context; in other words, behaviour was rarely tested in a setting in which multiple animals were able to interact freely. Automated behavioural scoring was the most common method for measuring behaviour (e.g. tools like Ethovision, ViewPoint, IDTracker), with 38.9% of articles using an automated quantification approach, 26.6% manually scoring behaviours from recordings, 21% using an indirect method of counting food consumption (e.g. counting food items remaining), and 8.6% used live scoring (all other methods were used in less than 1% of articles). It is important to highlight that 22.7% of articles (*n* = 221) did not clearly specify the methods used to measure behaviour; the information was considered as one of our validity indicators, and is also presented below in the validity assessment.

**Fig. 7:**
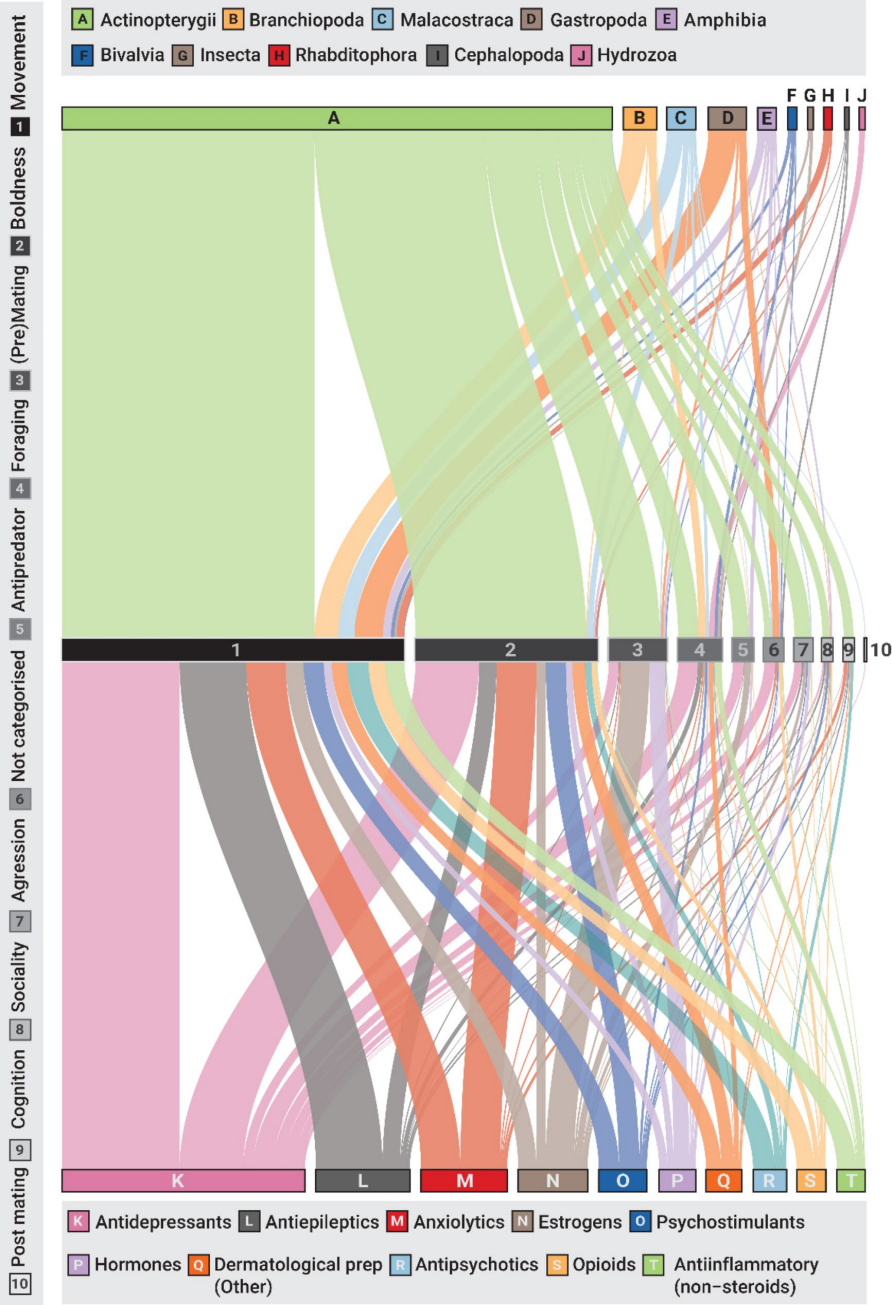
A broad overview of the link between population, exposure and outcome elements. The Sankey plot shows the connection between all behavioural categories (numbered 1–10; represented by the boxes in the middle of the plot), the top 10 most common phylogenetic clades (Class; shown at the top of the plot), and the top 10 therapeutic groups (ATC level 3; shown at the bottom of the plot). The thickness of each band that connects the population to behaviour, or exposure to behaviour element, corresponds to the number of occurrences in the EIPAAB database. An interactive version of the figure is available at https://jakemartinresearch.github.io/EIPAAB-database/

### Connecting population, exposure, and outcome (PEO)

Considering our population, exposure, and outcome elements (i.e. compounds, species, and behaviours) in combination, we found that most articles addressed the effect of a single pharmaceutical compound on a single species and measured a single behavioural category (41.5% of all articles). The next most common study design was a single pharmaceutical compound, a single species, and two behavioural categories (i.e. 17.7%), all other possible combinations each made up less than 10% of the articles. As a broad overview of the connections between compounds, species, and behaviours and how they varied, we illustrate below the links between the 10 most common phylogenetic clades (class) and each behavioural category, as well as the 10 most common therapeutic groups (ATC level 3; Fig. 7). Broadly speaking, for most of the top 10 clades, movement and locomotion are the most frequently measured behaviours, although there are clade-specific differences in the remaining behaviour categories. For example, Actinopterygii has a relatively high contribution to boldness behaviours, while Branchiopoda, Gastropoda, and Bivalvia are seldom used in the investigation of boldness-related behaviour (Fig. 7; see link in figure caption for an interactive version of the figure). There is even more variation in selected behavioural endpoints when looking at therapeutic groups. For example, antidepressants (ATC n06a), anxiolytics (ATC n03a), and psychostimulants (ATC n06b) have high relative contributions to measured boldness-related behaviour, while estrogens (ATC g03c) and hormones (l02a) have a high relative contribution to measured pre-mating/mating behaviour (Fig. 7). In the supplementary material, we further illustrate the variability in the relationship between compound, species, and behaviour using fluoxetine, diazepam, and 17-alpha-ethinylestradiol (the three most common compounds) as specific examples (see Additional File 3, Fig S7).

### Additional ecotoxicological endpoints

A secondary goal of our SEM was to collate information on additional endpoints (e.g. sub-organismal, reproduction, growth, survival) measured alongside behaviour to facilitate connections across domains that may be useful for future chemical risk assessment and management activities, including potential regulatory decision-making. We found that 51.7% of articles (466/901) also included at least one additional sub-organismal physiological or endocrine endpoint, such as hormone concentrations, biomarker expression, or mRNA transcription. In addition, 39.7% of articles (358/901) measured at least one endpoint that has been used in traditional ecotoxicity testing, such as survival, growth, reproductive output, or developmental abnormalities.

### Mapping the quality of the evidence base

Study validity was not used as an inclusion criterion; however, we did extract information about study validity to enrich the database and to identify potential methodological reporting gaps in the evidence base (all data relating to study validity are detailed in Additional File 7, in the ‘validity_assessment’ column). We extracted information relevant to a subset of study quality information from the CRED reporting guidelines [42] and several additional validity metrics (see Table 2 and Additional File 3, Table S3). To highlight key methodological and/or reporting gaps identified: we observed a low percentage of studies employing (or reporting) experimenter blinding during the scoring or analysing of behaviour (17.0%), randomly (or pseudo-randomly) assigning organisms to exposure treatments (40.2%), providing key details about the pharmaceutical compound used in the exposure (e.g. CAS registry number 24.8% or purity 25.4%), employing exposure concentration verification (e.g. water verification 20.6% or tissue verification 8.9%), following any type of guideline (or modified guideline; 15.0%), or performed the test under Good Laboratory Practice (GLP) conditions (0.7%). In the opposite direction, a high percentage of studies reported details related to the source of the animals (84.4%), aspects of animal care and housing (e.g. animal feeding 79.5%; water quality parameters 89.5%; dark-light cycle 83.9%), providing details about exposure duration (minimum exposure duration 94.1%, maximum duration 94.5%), and describing methods for scoring behavioural endpoints (77.3%; although we note lower levels of extractor consistency with some of these metadata; see Additional File 3, Table S6).

We should highlight that some of the species validity information may be implied or assumed to those with expert knowledge of that species; for example, if a species is hermaphroditic, sex may not have been reported; alternatively, for species that reach adulthood within 14 days, a 14-day exposure may have implied an adult life stage. With that said, we extracted these metadata based on the definitions given by the authors. Where information was not supplied, it was not assumed or inferred by extractors.

**Table 2:** All extracted information that relates to study validity. If the validity metadata are aligned with a CRED quality criteria [42], the associated CRED number is provided. The percentage of articles meeting the validity criteria is shown overall, and for each study motivation. NA indicated that the criterion was not part of CRED, but an additional criterion we extracted information about

Validity criteria	CRED	Overall	Environ	Medical	Basic
A guideline or modified guideline was followed	1	15%	21.8%	6.0%	6.4%
The test was performed under Good Laboratory Practice (GLP) conditions	2	0.7%	1.0%	0.4%	0.0%
Survival, growth and/or reproduction of the test organism(s) was reported	3	39.7%	53.5%	26.1%	15.3%
The test substance is identified with a CAS number	5	24.8%	36.7%	10/7%	7.0%
The purity of the test substance was reported	6	25.4%	38.8%	9.4%	5.7%
Organism(s) life stage is known and reported	8	83.4%	82.9%	91.0%	73.5%
Organism(s) sex is known and reported	8	53.5%	49.6%	59.0%	56.8%
Test organism source is reported	9	84.4%	86.9%	78.8%	84.5%
Information provided regarding feeding	11	79.5%	84.3%	68.2%	80.4%
Information provided regarding water characteristics (e.g. temperature, pH, oxygen content)	11	89.5%	92.7%	88.5%	80.3%
Information provided regarding light/dark conditions	11	83.9%	84.1%	85.9%	80.3%
Exposure minimum duration is defined	14	94.1%	95.6%	90.0%	96.7%
Exposure maximum duration is defined	14	94.5%	96.3%	89.8%	97.0%
The concentration of the test substance is verified in the water (waterborne exposures only)	15	20.6%	35.8%	2.5%	2.7%
The concentration of the test substance is verified in the tissue of the organism (waterborne exposures only)	15	8.9%	13.4%	4.2%	4.7%
Employs randomisation (pseudo-randomisation) of treatment allocation	NA	40.2%	44.9%	32.2%	36.7%
Experimental blinding was performed	NA	17.0%	14.7%	18.9%	21.5%
Methods for scoring behavioural endpoints described	NA	77.3%	76.0%	78.5%	79.4%
Conflict of interest statement is made in the article (with or without conflict identified)	NA	54.8%	50.2%	72.1%	44.3%

### Limitations of the systematic map

Two potential limitations of the evidence base to consider are the inherent complexity of assigning therapeutic classes to pharmaceuticals and the complexity of defining animal behavioural responses into discrete categories. First, we used Anatomical Therapeutic Chemical (ATC) Classification to group our compounds, which assigns active ingredients of drugs according to the organ or system on which they act and their therapeutic, pharmacological, and chemical properties [48]. However, it is well recognized, even by the World Health Organization (see “Classification Principles & Challenges” [51]), that pharmaceuticals can be prescribed and used for treating non-target illnesses. For example, beta-blockers (a family of blood-pressure regulating drugs) and certain antihistamines (used for treating allergies), can also be prescribed for the treatment of anxiety [52]. As a result of this complexity, we did not independently assign pharmaceuticals without an existing ATC class to their own therapeutic class. Thus, we highlight that 121 drugs (28% of the total database) are not included in summaries made at the pharmacological group level (e.g. Figure 5A). Similarly, it can also be complex to categorise animal behaviour into discrete overarching categories, as behaviour, and how scientists describe it, varies by species. Moreover, behaviour is context-dependent, in that a given behaviour measured in one context could represent a different underlying motivation in another context. For example, affiliation with a group of conspecifics may represent social propensity in one context but antipredator behaviour in another, if a perceived threat is present. We aimed to reduce ambiguity in assigning behaviours to overarching classes (and the sub-categories within each class) by following the author’s definition of the behaviour in the article. This could lead to inconsistencies where, for example, an animal solving a maze task could be defined as a measure of “boldness and exploration” in one article, but the same task could be a measure of “cognition” in another article. Moreover, authors can introduce inconsistencies even within articles if they define or refer to behaviours in multiple ways throughout the text. We note in the consistency section above that there was some extractor disagreement in the assignment of behavioural measures to the overarching categories, ranging from 75.5 to 99.3% (median 98.6%, see Table S6), as well as the more specific subcategories with a range of 67.6–99.3% (median 95.8%, see Table S6). We believe that this, in part, reflects the inherent difficulty of assigning behavioural classes across a broad range of taxa and study disciplines.

We also identified several potential limitations of the review search methods used. Although we included articles written in all languages in which our review team was proficient (8 different languages), the evidence is likely still biased towards research published in English, because the search strings were written in English, and there is a higher prevalence of English records in the databases used for the search. This is important to highlight as it is well recognized that language can introduce bias in the evidence base [53]. With that said, only 4 articles were excluded from the EIPAAB database at the full-text screening stage based on language. Another potential limitation in the review methods for this map is a limited search of the grey literature. Although we allowed for grey literature to be included from our database searches and we solicited grey literature submissions in our supplementary article search advertising calls, we did not search any grey literature databases and removed the planned screening of academic theses from the map. This decision was taken in part due to time and resources needed to screen the evidence base, but also because screening theses would require further quality checks and detailed deduplication cross-checks to remove duplicated published thesis chapters. We suggest this could be added for subsequent systematic review or meta-analytic projects using this database that have a narrower research scope. Finally, we also screened only a subset of articles at the full-text stage in duplicate, and we have discussed the implications of this above regarding extraction consistency.

## Conclusion

We sought to systematically synthesise all available Evidence for the Impacts of Pharmaceuticals on Aquatic Animal Behaviour (EIPAAB). We report a considerable amount of research on this topic, with 901 articles—representing over 1,700 behavioural assessments—being included in the EIPAAB database. Broadly, we see that the EIPAAB database would be ideal in supporting future ecotoxicology studies and experiments focusing on animal alternatives, identifying and incorporating evidence from behaviour endpoints into chemical risk assessment and management, to highlight knowledge gaps for future research, and to act as a launching pad for further targeted synthesis with more quantitative meta-analytical methodologies. The implications of the collated evidence for policy/management and research are discussed below.

### Implications for policy and management

Increasingly, behavioural endpoints are being suggested as valuable tools in environmental chemicals assessment and management (including regulatory activities for human and veterinary pharmaceuticals) but are rarely included in such context [17, 18]. There are several possible reasons for this, including poor reporting of methodology, using non-standard methods, and limited evidence in an ecotoxicological context of the links between behaviour and adverse outcomes/standard endpoints [54]. The EIPAAB database provides insights into all three of these potential barriers to inclusion in regulation.

Firstly, we have indeed identified several methodological and/or reporting pitfalls. This includes a lack of studies employing (or reporting) experimenter blinding during the scoring/analysing of behaviour, randomly (or pseudo-randomly) assigning organisms to exposure treatments, providing key information about the pharmaceutical compound used in the exposure (e.g. CAS registration number or purity), providing key information about the study organism used in the exposure (e.g. sex), and validating exposure concentration (e.g. water verification or tissue verification). Research on the effects of pharmaceuticals on animal behaviour would benefit from addressing these aspects of methodical reporting and study methodology, many of which require little additional effort from experimenters, and we hope that this review can be a catalyst to improve these aspects in the field. With that said, there are many articles that do not have these identified pitfalls in the evidence base, and if required, those seeking to use this evidence for regulatory purposes (or likewise) could filter the database to help identify those studies that meet relevant criteria. More broadly, the field of behavioural ecotoxicology and toxicology studies with animal alternatives (e.g. fish models) could benefit from the use of data reporting and reliability guidelines specific to behavioural endpoints to increase the likelihood of these studies being included in future chemical risk assessment and management, such as regulatory processes. A recent set of such guidelines is provided in EthoCRED ( [55]), a behavioural endpoint-specific adaptation of the parent CRED guidelines. The use of such guidelines, like EthoCRED, would improve reporting of important methodological information, guide methodological decision-making for future studies, and increase the replicability of the field.

Secondly, the database included a total of 63 different sub-categories of measured behaviours and for which aquatic species they were measured. From these data, we suggest that new standardised ecotoxicity test guidelines that include behaviour could be developed by looking for the most common or most widely applicable testing parameters. As an example, our SEM has revealed a wealth of studies focused on fishes (especially for zebrafish) across multiple behavioural endpoints (particularly movement, anxiety/boldness, and pre-copulatory/copulatory behaviours); by comparing such methods, one could arrive at the most broadly suitable tests. We believe that the next step in achieving this would be a focused review and meta-analysis, looking at the specific methods used for candidate behavioural tests and the nature of the data they provide, followed by multi-lab validity and repeatability tests once a candidate protocol is established.

Thirdly, within the EIPAAB database, we have identified which studies can provide direct links between behaviour and other adverse outcomes/standard endpoints. Specifically, we have identified studies that also measured sub-organismal physiological/endocrine endpoints (*n* = 466; 51.7%), as well as studies that assessed more traditional endpoints like animal growth, survival, and/or reproduction alongside behaviour (*n* = 358; 39.7%). We see this as a starting point for future work to connect behavioural endpoints to molecular initiating events and to endpoints currently being used in traditional risk assessments, including integration with the adverse outcome pathway (AOP) concept [56]. Specifically, we suggest targeted meta-analytic approaches focusing on articles that have measured behaviour alongside additional morphometric endpoints (sub-organismal, growth, survival, and/or reproduction endpoints), identifying potential correlations in the direction and magnitude of observed effects.

### Implications for research

Our SEM highlights that this rapidly growing research area has several knowledge clusters appropriate for further quantitative synthesis. Specifically, future meta-analytical work could focus on the behavioural impacts of antidepressants, antiepileptics, or estrogens, particularly for endpoints like locomotion, boldness, and reproductive behaviours. We have also identified that the evidence base is heavily skewed towards research on zebrafish, which is perhaps unsurprising given that the zebrafish is a well-established model in (eco)toxicological, medical, and basic research [57, 58]. Therefore, future comparative synthesis across behavioural categories or compounds using zebrafish may offer a suitably homogenous prospect for detailed meta-analysis. Indeed, the available evidence on zebrafish could be a valuable step towards disentangling and identifying quantitative thresholds at which exposure to a given pharmaceutical affects behaviour. For instance, how, and at which exposure concentration, the antidepressant fluoxetine impacts fish behaviour has been disputed in the earlier literature [44]. We would also like to highlight gaps in the evidence base that require more primary research. Firstly, there were relatively few studies using wild-caught animals. Wild-caught *versus* lab-reared organisms can differ greatly in their behaviour and underlying physiology traits [59–62], and thus, may also respond differently to pharmaceutical exposure. More research using wild-caught organisms could help identify whether lab-reared model species are equally sensitive to pharmaceutical exposure (e.g [63]). Additionally, locomotion and boldness were by far the most common behavioural endpoints measured. We argue that measuring contaminant-induced impacts on a more diverse array of behavioural endpoints—particularly those with obvious links to fitness (e.g. pre and post-copulatory, antipredator, and foraging behaviours)—would give a more holistic understanding of potential impacts on aquatic wildlife. However, we also acknowledge that the most commonly measured behaviours, locomotion and boldness, are often the simplest to measure and offer the highest throughput. There was also a distinct lack of studies measuring behaviour within a social context (e.g. free-swimming groups) and employing exposure durations greater than a week; it is reasonable to assume that for most animals, real-world exposures will occur in social groups (animals rarely, if ever, exist in a social vacuum; [64]), and that many pollutants would have environmental or biological half-lives exceeding seven days. Thus, future research addressing the impacts of pharmaceutical pollutants on animals under a social context and over chronic time scales would improve our understanding of real-world impacts. Finally, we suggest that research is prioritised on pharmaceutical compounds that are absent or infrequently represented in our database, yet are common in the environment (i.e. what evidence are we currently missing). This could be done by cross-checking the EIPAAB database against recent publications (e.g [1]). and open databases reporting environmental pharmaceutical concentrations around the world (e.g. AstraZeneca EcoPharmacoVigilance Dashboard [65]; Umwelt Bundesamt “UBA-PHARMS” database [66]; NORMAN EMPODAT chemical occurrence database [67]).

We identified that many of the studies in our database have an environmental motivation; however, we also identified a lot of available research in adjacent fields that focus on medical research questions and basic research questions, particularly with fish models employed as animal alternatives. Future work assessing the bibliometric connections between the fields would be interesting to reveal how much crosstalk (if any) exists via the use of co-author and co-citation networks [34].

We have already pointed out several gaps in study validity that should be considered in future studies and noted that using standard reporting guidelines would increase their utility in regulatory processes. We also advocate that the use of reporting guidelines (e.g. EthoCRED) will more broadly increase the robustness and replicability of studies assessing the effects of pharmaceuticals on aquatic animal behaviour. Importantly, we highlight that disclosing details about how animals were housed, how they were assigned to treatments, how the behaviour was recorded and scored, and the use of blind scoring, is paramount to increasing transparency and reducing unintended experimenter bias.

## Electronic supplementary material

Below is the link to the electronic supplementary material.

Additional file 1: ROSES Form (name: martin-et-al-additional-file-1-ROSES.xlsx; link: https://osf.io/vwz3m)

Additional file 2: Full-text screening and extraction form, made in Qualtrics (name: martin-et-al-additional-file-2-full-text-screening-extraction-form.pdf; link: https://osf.io/w6kjr).

Additional file 3: Supplementary materials for the article (name: martin-et-al-additional-file-3-supplementary-materials.pdf; link: https://osf.io/m7s3z).

Additional file 4: Title and abstract screening decisions (name: martin-et-al-additional-file-4-title-abstract-screen-decision.xlsx; link: https://osf.io/fy7xp).

Additional file 5: List of eligibility disagreements for duplicate screenings at the full-text screening stage (name: martin-et-al-additional-file-5-eligibility-disagreements.xlsx; link: https://osf.io/cyauf).

Additional file 6: Full-text screening excluded articles (name: martin-et-al-additional-file-6-full-text-exculded-atricles.xlsx; link: https://osf.io/qwjby).

Additional file 7: Read me file for the database (name: martin-et-al-additional-file-7-database-READ-ME.xlsx; link: https://osf.io/2h8jg)

Additional file 8: R script used to summarise the EIPAAB Database interactive HTLM (https://jakemartinresearch.github.io/EIPAAB-database/); a static version is also available on OSF (name: martin-et-al-additional-file-8-r-script.Rmd; link: https://osf.io/2wc7f).

Additional file 9: The ROSE flow diagram (name: martin-et-al-additional-file-9-ROSES-diagram.pdf; link: https://osf.io/pxu5y).

Additional file 10: The Web of Science annual article counts for each of the most common research categories identified in the database (name: martin-et-al-additional-file-10-wos-research-areas-1992-2022.xlsx; link: https://osf.io/t8yja).

## Data Availability

Availability of data and materials: All supplementary files and the EIPAAB database can be accessed from the Open Sciences Framework (OSF) at https://doi.org/10.17605/OSF.IO/ATWY6. Below, we provide a list of all supplementary files and individual links. The R script used to generate the summary statistics and figures presented in this manuscript is available on GitHub (https://github.com/JakeMartinResearch/EIPAAB-database).
