# Supplementary material for: Evidence of the impacts of pharmaceuticals on aquatic animal behaviour (EIPAAB): a systematic map and open access database
Source: Environ Evid. 2025 Mar 20;14:4. doi: 10.1186/s13750-025-00357-6 (PMC11924672; doi:10.1186/s13750-025-00357-6)
Supplement: Supplementary file 7 — Additional file 7: Read me file for the database (name: martin-et-al-additional-file-7-database-READ-ME.xlsx; link: https://osf.io/2h8jg) [file 13750_2025_357_MOESM7_ESM.pdf]

Full text screening - Systematic map final

Start of Block: Introduction

Q1 **Systematic Map - Full text screening**

This form includes the full-text screening and meta-data extraction.

1) You will have been given a list of papers to extract.

2) Download and save the paper as a PDF using its Paper ID as the file name and your initials. Use the highlighting and commenting function in your PDF viewer to highlight information in the manuscript relevant to the answers in this google form. This will be helpful later if you need to discuss where you found details with other extractors. It's also good practice.
"{Paper ID}_{INTIALS}.PDF"

3) Use the decision trees we've made to guide you on when to exclude a paper and what constitutes a compound of interest (FAQ doc). If you are uncertain about whether a paper should be included or excluded, there is an option at the end to "Discuss" the paper with others before you make your inclusion/exclusion decision (but you will still extract all the meta-data).

4) Before starting your extraction, consider if your paper has 1) multiple animal species (more than 5 will require you to complete the form twice) or 2) multiple exposure compounds of interest (more than 10 will require you to complete the form twice). It's important you remember what you have done on which form.

5) Use the "Comments" section at the end to elaborate on any specific question answers that caused problems - remember to write down the question number you're referring to!

6) Save your answers at the end as a PDF, you will automatically be given this option.

NOTE: You can leave this survey when you are partially done and extraction and return to it. But, after you finish and submit an extraction, you cannot *easily* return to it. If you need to change any of your answers, you will have to ask Erin/Jake to email you an editing link.

End of Block: Introduction

Start of Block: Article and screener information

Q2 **Screener initials**

First, middle and last, with no periods between the letters, e.g. ESM, JMM

____

Q3 **Article ID**

You'll find the Article ID in the paper list file you've been provided.

____

Q4 **DOI**

Formatted with NO spaces. Formatted without URL at start e.g. 10.3923/jfas.2015.111.120 ...NOT https:/doi.10.3923.... If no DOI, put "NA"

____

Q5
Extractions
Is this the first form used to extract this paper?

○ Yes (1)

○ No (2)

End of Block: Article and screener information

Start of Block: Inclusion criteria

Q6 **Inclusion criteria**

These are the key questions that evaluate whether the paper should be included or excluded in

the map. Answer all of them fully because we want to know if the paper was excluded for multiple reasons. If we are asked later why a paper was excluded, we will refer to these answers.

Q7 **Animal class**
What type of organism is used to study impacts on behaviour? Select all that apply. At least one aquatic organism must be studied for the paper to be included.

- ☐ Fish (1)
- ☐ Aquatic invertebrate (2)
- ☐ Amphibian (3)
- ☐ Reptile (4)
- ☐ Aquatic mammal (5)
- ☐ No aquatic animal (6)

Q8 **Animal strain/genetically modified**
Are the animals used in the paper genetically modified strains (e.g. mutant, receptor knock-out, transgenic)? At least one set of "natural" animals (i.e. control and treatment groups) must be included. NOTE: multiple lines (including both short- and long-fin) of zebrafish are considered wild type. See: https://zfin.org/action/feature/wildtype-list.

- ○ Natural or wild type (1)
- ○ Assume natural or wild type (2)
- ○ Only genetically modified (3)

Q9 **Are organisms exposed to a compound of interest? (single exposure)**
We include exposures with at least one compound of interest in a single exposure setting, meaning no papers using only chemical mixture exposures. See FAQ document for guidance.

- ◯ Yes (1)
- ◯ No (2)
- ◯ Uncertain (3)

Q10 **Is there a control group?**
It does not matter what type of control is included (e.g. solvent control vs freshwater control). Therefore, the article is also not a review, conference proceeding, meta-analysis.

- ◯ Yes (1)
- ◯ No (2)

Q11 **Is behaviour measured?**
Is at least one behavioural endpoint measured?

- ◯ Yes (1)
- ◯ No (2)

Q12 **Publication language**

- ◯ English (1)
- ◯ Czech (3)
- ◯ Japanese (6)
- ◯ Norwegian (5)
- ◯ Polish (8)
- ◯ Russian (9)
- ◯ Slovak (7)
- ◯ Swedish (2)
- ◯ Other (4) ____

Q13 **Should this paper be included or excluded?**
Based on your answers to these questions and the decision tree, choose whether this paper should be included and excluded for meta data extraction. If you choose "Discuss" you will still extract the meta-data.

- ◯ Include (1)
- ◯ Exclude (2)
- ◯ Discuss (3)

Display This Question:

If Q13 = Exclude

Q14 **Elaborate on the reason for exclusion**
If you chose "exclude", please elaborate on the specific reasoning behind your choice to exclude.

____

Skip To: End of Survey If Condition: Elaborate on the reason for... Is Not Empty. Skip To: End of Survey.

Display This Question:

If Q13 = Discuss

Q15 **Elaborate on the reason for discussion**
If you chose "discuss", please elaborate which details need to be discussed.

____

End of Block: Inclusion criteria

Start of Block: How many species

Q16 **How many species of interest are used in behavioural measures?** (maximum of 5 can be extracted with one form)

____

End of Block: How many species

Start of Block: Study species

Q17 **What is the scientific name of species ${lm://Field/1}?**
E.g. Neogobius melanostomus, Salmo trutta. Do NOT use the common name.

____

Page Break

Q18 **What is the source of ${Q17/ChoiceTextEntryValue} used in this experiment?**
A commercial supplier could be a breeder, hatchery, pet or aquarium store. "Lab stock" refers to animals that were not caught or purchased for the purpose of this experiment and were already in the lab for some amount of time before being used (could refer to a single or multiple generation(s)).

- ☐ Wild collected (1)
- ☐ Commercial supplier or fish farm (2)
- ☐ Lab stock from wild population (3)
- ☐ Lab stock from commercial supplier (4)
- ☐ Lab stock of undisclosed origin (7)
- ☐ Not reported (5)

Q19 **At what life stage was behaviour measured for ${Q17/ChoiceTextEntryValue}?**
Select all that apply if behaviour is measured on multiple life stages. Tadpoles are included as larvae. Fry are included as juvenile. Larvae have emerged from the yolk sac and are therefore external from the embryo or the mother.

- ☐ Egg or embryo (1)
- ☐ Larvae (7)
- ☐ Juvenile (3)
- ☐ Adult (4)
- ☐ Unknown or not specified (6)

Q20 **What sex(es) are used in the behavioural testing following exposure for ${Q17/ChoiceTextEntryValue}?**
Follow what the authors say. i.e., they must say it is hermaphroditic for you to select that option. Select all that apply.

- ▢ Male (1)
- ▢ Female (2)
- ▢ Hermaphrodites (3)
- ▢ Unknown or not specified (6)

End of Block: Study species

Start of Block: How many compounds

*

Q21 **How many different compounds of interest were used?** (maximum of 10 per form)

____

End of Block: How many compounds

Start of Block: Compounds, Exposure and Behaviour

Q22 **What was the name of compound ${lm://CurrentLoopNumber}?**
Capitalize the first letter when appropriate, eg., Fluoxetine, Oxazepam, 17β-estradiol

____

Skip To: Q23 If Condition: What was the name of compou... Is Not Empty. Skip To: Is the CAS identification code given ....

Q23 **Is the CAS identification code reported for ${Q22/ChoiceTextEntryValue}?**
 Authors need to specifically state that it is a CAS code in the text and not some other form of product identifier from Sigma or Merch etc.

- ◯ Yes (3)
- ◯ No (1)

Q24 **Is the purity of ${Q22/ChoiceTextEntryValue} reported (i.e. before any exposure doses were made)?** Purity is usually reported as a percentage.

- ◯ Yes (1)
- ◯ No (2)

Q25 **What was the primary route of exposure with the compound ${Q22/ChoiceTextEntryValue}?**
 Other exposure routes include foodborne, sediment, injection or implant.

- ◯ Waterborne only (1)
- ◯ Waterborne plus any other route (5)
- ◯ Other exposure route (4)

Skip To: Q26 If Q25 = Waterborne only

Skip To: Q26 If Q25 = Waterborne plus any other route

Skip To: Q32 If Q25 = Other exposure route

---

*

Q26 **How many waterborne exposure groups are there for the behavioural experiment with ${Q22/ChoiceTextEntryValue}, INCLUDING the control?**

____

Q27 **What is the dose for the LOWEST waterborne exposure group for the behavioural experiment with ${Q22/ChoiceTextEntryValue} (that is not the control)?**
Use the measured concentration if it was quantified in some way. If multiple measured values are given (e.g., over multiple replicates) give the lowest value. If only the mean is given, use the mean.

____

Q28 **What are the units of the concentration of the LOWEST waterborne dose for the behavioural experiment with ${Q22/ChoiceTextEntryValue}?**

○ pg/L (1)

○ ng/L (2)

○ ug/L (3)

○ mg/L (4)

○ g/L (5)

○ nM (6)

○ uM (7)

○ mM (8)

○ Other (9) ____

---

*

Q29 **What is the dose for the HIGHEST waterborne exposure group for the behavioural experiment with ${Q22/ChoiceTextEntryValue}?**
Use the measured concentration if it was quantified in some way. If multiple measured values are given (e.g., over multiple replicates) give the lowest value. If only the mean is given, use the mean.

____

Q30 **What are the units of the concentration of the** HIGHEST waterborne **dose for the <u>behavioural experiment</u> with ${Q22/ChoiceTextEntryValue}?**

○ pg/L (1)

○ ng/L (2)

○ ug/L (3)

○ mg/L (4)

○ g/L (5)

○ nM (6)

○ uM (7)

○ mM (8)

○ Other (9) ____

Q31 **Are the waterborne exposure concentrations for ${Q22/ChoiceTextEntryValue} measured and validated <u>from the behavioural experiment</u>?**
For example, is it measured via some form of analytical chemistry or assay (e.g. LCMS, GCMS, ELISA etc.). They must be measured during the behavioural experiment itself. See FAQ document for further details.

○ Measured (1)

○ Nominal (2)

Q32 **What was the <u>minimum</u> exposure duration before a behavioural assay?**
If only one exposure duration was used, enter that duration for both min (previous question) and max. Do not make assumptions about duration, if it is not stated, select that option.

Lifetime if the organism is exposed from birth and an exact duration is not given.

Multigenerational if parents were exposed and the focal animals were still being exposed. Transgenerational if the parents were exposed but the focal animals were raised in a clean environment.

- ○ < 6 h (2)
- ○ > 6 h to ≤ 24 h (3)
- ○ > 1 d to ≤ 3 d (4)
- ○ > 3 d ≤ 8 d (5)
- ○ > 8 to ≤ 15 d (6)
- ○ > 15 d to ≤ 22 d (7)
- ○ > 22 d to ≤ 29 d (8)
- ○ > 29 d to ≤ 3 month (9)
- ○ > 3 month to 6 month (13)
- ○ Lifetime (12)
- ○ Multigenerational (10)
- ○ Transgenerational (11)
- ○ Not stated (14)

Q33 **What was the maximum exposure duration before a behavioural assay?**
If only one exposure duration was used, enter that duration for both min (previous question) and max. Do not make assumptions about duration, if it is not stated, select that option.

Lifetime if the organism is exposed from birth and an exact duration is not given.
Multigenerational if parents were exposed and the focal animals were still being exposed.

Transgenerational if the parents were exposed but the focal animals were raised in a clean environment.

- ◯ < 6 h (2)
- ◯ > 6 h to ≤ 24 h (3)
- ◯ > 1 d to ≤ 3 d (4)
- ◯ > 3 d ≤ 8 d (5)
- ◯ > 8 to ≤ 15 d (6)
- ◯ > 15 d to ≤ 22 d (7)
- ◯ > 22 d to ≤ 29 d (8)
- ◯ > 29 d to ≤ 3 month (9)
- ◯ > 3 month to 6 month (13)
- ◯ Lifetime (12)
- ◯ Multigenerational (10)
- ◯ Transgenerational (11)
- ◯ Not stated (14)

Q34 **Where were animals exposed to ${Q22/ChoiceTextEntryValue}?**
Outdoor restricted could be net- or land-based mescosms, net-pens, ponds. Outdoor natural setting means animal is free to move about and may easily interact with other aquatic organisms not the focus of the study.

- ◯ Indoor laboratory setting or assumed indoors (1)
- ◯ Outdoor restricted setting (cannot interact with wild species) (3)
- ◯ Outdoor natural setting (4)

Page Break

Q35
Behaviour measured for exposure to ${Q22/ChoiceTextEntryValue}.

When extracting behaviour, focus on what assay(s) the authors use to measure behaviour. Do not get hung-up on the specific endpoints or the units that they used in each assay. Follow the way the authors operationalize the behavioural assay in each paper. For example, if they use a maze to study cognition, then call it "cognition" or if they use a maze to study boldness, then call it "boldness".

Q36 **Movement and Activity**
How was activity or movement assayed? Select all the apply. If the assay is not broadly captured by one of the provided options, provide a definition in "Other". Abnormal movement can include seizures.

- ☐ Normal locomotor activity (1)
- ☐ Abnormal movement (2)
- ☐ Dispersal (3)
- ☐ Migration (4)
- ☐ Other (5) ____

Q37 **Courtship and Mating**
How was courtship and/or mating behaviour assayed? Select all the apply. Pre-copulatory behaviour includes courtship movements, displays, guarding, following, pursuing etc. Focus on

the setup when behaviour was being recorded. If the assay is not broadly captured by one of the provided options, provide a definition in "Other".

- ▢ Nest-building preparation or acquisition (1)
- ▢ Mate choice or preference (between some number of options) (2)
- ▢ Pre copulatory behaviour towards a model or video (3)
- ▢ Pre copulatory behaviour towards a conspecific behind a barrier (4)
- ▢ Pre copulatory behaviour and/or actual mating with a conspecific (free to interact) (5)
- ▢ Locomotor activity within this context (7)
- ▢ Other (6) ____

Q38 **Post-mating and parental care**
How were post-copulatory and/or parental care behaviours assayed? Select all the apply. If the assay is not broadly captured by one of the provided options, provide a definition in "Other".

- ▢ Resource defence (mate nest or offspring guarding/defence from a threat) (1)
- ▢ Offspring care (eg feeding cleaning fanning carrying nest tending lure displays) (2)
- ▢ Offspring cannibalism (3)
- ▢ Locomotor activity within this context (5)
- ▢ Other (4) ____

Q39 **Aggression**
How was conspecific aggression assayed? NOTE: distinguish between aggression in a

reproductive or predation context. Select all the apply. If the assay is not broadly captured by one of the provided options, provide a definition in "Other".

- ☐ Aggression towards a mirror (1)
- ☐ Aggression towards a model or video conspecific (2)
- ☐ Aggression towards a live conspecific behind a barrier (3)
- ☐ Aggression towards a live conspecific where both are free to interact (4)
- ☐ Locomotor activity within this context (6)
- ☐ Other (5) ____

Q40 **Sociality**
How was sociality assayed? Select all the apply. If the assay is not broadly captured by one of the provided options, provide a definition in "Other".

- ☐ Social choice (between some number of options) (1)
- ☐ Affiliation with a conspecific(s) model or video (2)
- ☐ Affiliation with a live conspecific(s) behind a barrier (3)
- ☐ Affiliation with a live conspecific(s) where they are free to interact (4)
- ☐ Locomotor activity within this context (6)
- ☐ Other (5) ____

Q41 **Cognition and learning**
How was cognition assayed? Select all the apply. If the assay is not broadly captured by one of the provided options, provide a definition in "Other".

- ☐ Avoidance learning (negative stimuli eg predator or noxious cues) (1)
- ☐ Associative learning (positive stimuli eg food or conspecific eg conditioned place preference) (2)
- ☐ Social learning from a knowledgeable conspecific(s) (3)
- ☐ Novel task problem solving (eg maze or puzzle box) (4)
- ☐ Memory assay (testing a learned association after time as passed) (5)
- ☐ Habituation task (desensitisation to a stimulus) (6)
- ☐ Locomotor activity within this context (8)
- ☐ Other (7) ____

Q42 **Boldness or anxiety**
How was boldness or anxiety assayed? Select all the apply. If the assay is not broadly captured by one of the provided options, provide a definition in "Other". NOTE - difference between black-white, and light on-off assays! For light on-and-off the animals do not shuttle back and

forth between compartments, a light is turned on and off over the same arena. Scototaxis (black white, light dark) the animals shuttle back and forth between compartments.

- ☐ Black-white arena (ie scototaxis or light-dark) (1)
- ☐ Light on-off (in one arena and a light is turned on and off) (2)
- ☐ Novel tank or novel tank diving or exploration or open-field (3)
- ☐ Novel object (8)
- ☐ Emergence from shelter or hiding (from a place of relative safety) (4)
- ☐ Shoaling (ie tighter shoals or increased anxiety) (5)
- ☐ Locomotor activity within this context (7)
- ☐ Other (6) ____

Q43 **Foraging**

How was foraging or feeding assayed? Select all the apply. If the assay is not broadly captured by one of the provided options, provide a definition in "Other"

- ☐ Foraging on a live food source (1)
- ☐ Foraging on a not live food source (2)
- ☐ Locomotor activity within this context (4)
- ☐ Other (3) ____

Q44 **Antipredator**
How were antipredator responses assayed? Select all the apply. If the endpoint or assay is not broadly captured by one of the provided options, provide a definition in "Other".

- ☐ Response to a simulated predator (eg marble drop or model or chasing) (1)
- ☐ Response to a predation olfactory cue (predator and conspecific alarm cues) (2)
- ☐ Response to a live predator behind a barrier (3)
- ☐ Response to a live predator where animals are free to interact (4)
- ☐ Locomotor activity within this context (6)
- ☐ Other (5) ____

Q45 **Other behaviour**
If another behaviour was assayed but is not described above, state what it was here.

____

Page Break

Q46 **Is behaviour measured in a social context following exposure to ${Q22/ChoiceTextEntryValue}?**
Are any of the behaviours being measured in the presence of a free-swimming conspecific (the animals can freely interact with no barrier)? The conspecific can be exposed or unexposed, and it does not matter if the conspecific's behaviour was scored or not.

○ Yes (1)

○ No (2)

Q47 **In what setting was the behaviour measured during exposure to ${Q22/ChoiceTextEntryValue}?**
Select all that apply. Note this can be different than the exposure location.

☐ Indoor laboratory setting or assumed indoors (1)

☐ Outdoor restricted setting (cannot interact with wild species) (3)

☐ Outdoor natural setting (4)

Q48 **Were internal concentrations of ${Q22/ChoiceTextEntryValue} measured in animal tissues or plasma at any time during the study?**
This can include animals that did not have behaviour tested on them.

○ Yes (1)

○ No (2)

End of Block: Compounds, Exposure and Behaviour

Start of Block: Interactive treatments & Connecting across biological scales

Q49 **Compound mixtures**
Were animals also exposed to any compound mixtures in this paper?

- ◯ Yes (1)
- ◯ No (2)

Q50 **Are any sub-organismal physiological or endocrine traits/biomarkers measured?**
This can include many different things: hormone concentrations, neurotransmitter concentrations, receptor-related traits (density, expression), enzyme activity (eg CYPs, EROD), vitellogenin concentrations, sperm traits, fecundity, tissue histology, epigenetic endpoints, metabol-, proteon-, or transcript-omics, mRNA transcript abundance (gene expression). This can also be included if a different subset of animals than behaviour was measured on, as long as they received the same exposure.

- ◯ Yes (1)
- ◯ No (2)

Q51 Is survival, growth, or any components of reproductive success also measured?
This can include things like survival by treatment, growth, developmental abnormalities, morphological abnormalities, offspring fertilized or produced, parentage, offspring reared to independence etc. This can also include a different subset of animals than behaviour was measured on, as long as they received the same exposure.

- ◯ Yes (9)
- ◯ No (10)

End of Block: Interactive treatments & Connecting across biological scales

---

Start of Block: Study validity

Q52 **Did any part of this study follow a guideline method or modified guideline method (e.g. OECD, ISO)?**

This can include following a guideline method during the exposure phase and then adding behavioural measures beyond the guideline.

- Yes (1)
- No (2)

Q53 **Is this study performed following "Good Laboratory Practice" (GLP)?**
It must be specifically stated, see https://www.oecd.org/chemicalsafety/testing/overview-of-good-laboratory-practice.htm for details.

- Yes (1)
- No (2)

Q54 **Is information about animal feeding provided?**
This may include a schedule, or justification for feeding (or not feeding), as long as the details are given.

- Yes (1)
- No (2)

Q55 **Is the light-dark cycle described?**
Also select "Yes" if the experiment was conducted outdoors or they describe that ambient/natural lighting was used.

- Yes (1)
- No (2)

Q56 Are water quality parameters reported at any stage of the **experiment**?
For example, any of the following: temperature, pH, hardness, dissolved oxygen, ammonia. All parameters apply to the water (i.e. not the air). This could include numbers or just a verbal

description (e.g. temperature and pH were with normal limits for this species throughout the exposure).

○ Yes (1)

○ No (2)

Q57 **Treatment randomization?**
Are animals assigned to treatment groups randomly or haphazardly (this can include counterbalancing to account for other factors like sex)? "No" means it is clear and explained why animals were not randomly assigned. "Not disclosed" means there is not mention of how animals were assigned to treatment.

○ Yes (1)

○ Not disclosed (3)

○ No (2)

Q58 **How was behaviour scored for the ${Q22/ChoiceTextEntryValue} exposure?**
Select all that apply. If "Other" please elaborate. Try not to make assumptions about how behaviour was scored. If they do not specify, then select "not specified".

▢ Live-scoring in real-time (1)

▢ Manual or human scoring from videos (eg including use of JWatcher or Boris or non-automated Ethovision) (2)

▢ Supervised automated approaches (eg Ethovision or Zebrabox or ToxTrac) (3)

▢ Not specified (5)

▢ Other (6) ____

Q59 **Blinded behavioural scoring**
Are behaviours being scored blind to exposure treatment? The authors still must write that it was scored blind, even if behaviours are scored automatically (ethovision, zebrabox). If it is not mentioned, then you select. "Not disclosed" means there is no mention of whether scoring was blind or not. "No" means it is clear and explained thats scoring was not blinded.

○ Yes (1)

○ No (2)

○ Not discolsed (3)

Q60 Conflict or **competing** interests statement
Do the authors have a conflict or competing interests statement? If so, do they declare a conflict? This can be phrased as competing financial interests.

○ No conflict stated (1)

○ Some conflict stated (2)

○ No statement is made in the paper (3)

End of Block: Study validity

Start of Block: Motivation

Q61 **Primary motivation**
What is the main motivation of the paper? Extract this from reading the abstract, introduction/hypotheses, discussion/conclusions, and look at the journal name. If "Other" please elaborate.

○ Environmental (to understand how anthropogenic pollution affects animals) (1)

○ Medical (to understand human illness or processes via animal models) (2)

○ Basic research (to understand fundamental physiology or neurochemistry or behaviour) (3)

○ Other (4) ____

End of Block: Motivation

Start of Block: Final questions

Q62 **Elaboration and comments**
Here you can also leave any comments, concerns, thoughts about the extraction process not captured above. Please include the question number that your concern or uncertainty applies to!

____

End of Block: Final questions
