# Supplementary figures and images for: Evidence of the impacts of pharmaceuticals on aquatic animal behaviour (EIPAAB): a systematic map and open access database
Source: Environ Evid. 2025 Mar 20;14:4. doi: 10.1186/s13750-025-00357-6 (PMC11924672; doi:10.1186/s13750-025-00357-6)

# ROSES Flow Diagram for Systematic Reviews

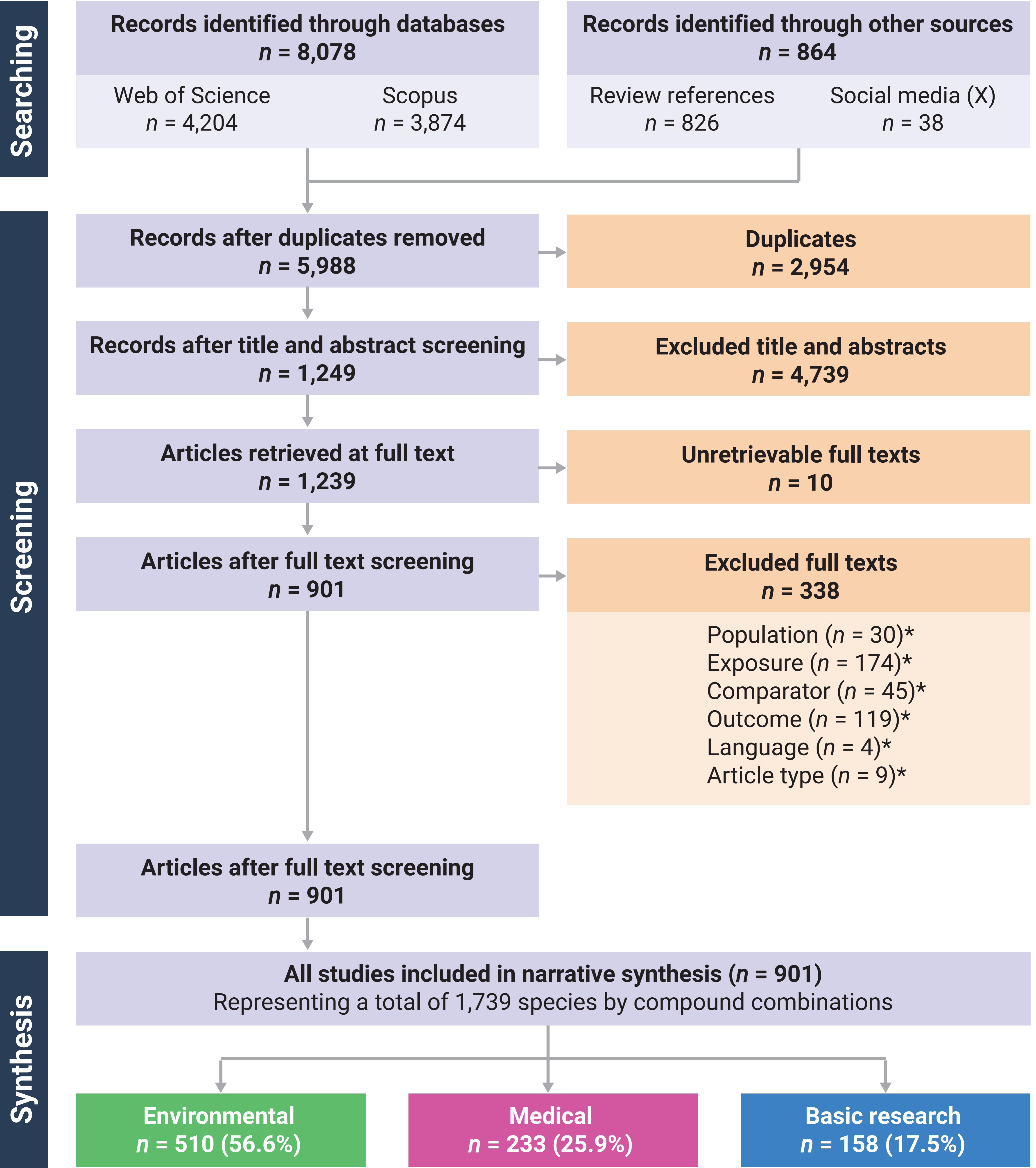

Supplement: Supplementary file 8 — Additional file 8: R script used to summarise the EIPAAB Database interactive HTLM (https://jakemartinresearch.github.io/EIPAAB-database/); a static version is also available on OSF (name: martin-et-al-additional-file-8-r-script.Rmd; link: https://osf.io/2wc7f). [file 13750_2025_357_MOESM8_ESM.pdf]
