# Supplementary material for: Evidence of the impacts of pharmaceuticals on aquatic animal behaviour (EIPAAB): a systematic map and open access database
Source: Environ Evid. 2025 Mar 20;14:4. doi: 10.1186/s13750-025-00357-6 (PMC11924672; doi:10.1186/s13750-025-00357-6)
Supplement: Supplementary file 9 — Additional file 9: The ROSE flow diagram (name: martin-et-al-additional-file-9-ROSES-diagram.pdf; link: https://osf.io/pxu5y). [file 13750_2025_357_MOESM9_ESM.pdf]

Evidence of the Impacts of Pharmaceuticals on Aquatic Animal Behaviour (EIPAAB): a systematic map and open access database

Additional File 1 - Supplementary Materials

Contents

Contents 1
Additional Files: Links for all additional files **3**
Supplementary Table S1: Changes to the full-text screening and data extraction form 5
Supplementary Table S2: Full search strings used in Web of Science and Scopus 14
Supplementary Table S3: A list of CRED criteria and details 16
Supplementary Table S4: Behavioral categories and definitions 18
Supplementary Table S5: Behavioral scoring methods 23
Supplementary Table S6: Metadata consistency estimates 24
Supplementary Table S7: Species metadata summary 26
Supplementary Figure S1: Compound decision tree 27
Supplementary Figure S2: Distribution of species use 28
Supplementary Figure S3: Taxonomic use by study motivation 29

Supplementary Figure S4: Number of compounds used per study 30
Supplementary Figure S5: Therapeutic class level one 31
Supplementary Figure S6: Behavioural sub-categories 32
Supplementary Figure S7: Example links between population, exposure, outcome 34

Additional files: Links for all additional files

(1) **EIPAAB database:** the Evidence of the Impacts of Pharmaceuticals on Aquatic Animal Behaviour (EIPAAB) database created from the systematic review and data extraction described in the manuscript (name: EIPAAB-database.csv; link: https://osf.io/rzwv2 OR https://github.com/JakeMartinResearch/EIPAAB-database) * To avoid confusion when downloading the database this is not named as an 'Additional File')

(2) **Additional file 1:** ROSES Form (name: martin-et-al-additional-file-1-ROSES.xlsx; link: https://osf.io/vwz3m)

(3) **Additional file 2:** Full-text screening and extraction form, made in Qualtrics (name: martin-et-al-additional-file-2-full-text-screening-extraction-form.pdf; link: https://osf.io/w6kjr)

(4) **Additional file 3:** Supplementary materials for the article (name: martin-et-al-additional-file-3-supplementary-materials.pdf; link: https://osf.io/m7s3z)

(5) **Additional file 4:** Title and abstract screening decisions (name: martin-et-al-additional-file-4-title-abstract-screen-decision.xlsx; link: https://osf.io/fy7xp)

(6) **Additional file 5:** List of eligibility disagreements for duplicate screenings at the full-text screening stage (name: martin-et-al-additional-file-5-eligibility-disagreements.xlsx; link: https://osf.io/cyauf)

(7) **Additional file 6:** Full-text screening excluded articles (name: martin-et-al-additional-file-6-full-text-exculded-atricles.xlsx; link: https://osf.io/qwjby)

(8) **Additional file 7:** Read me file for the database (name: martin-et-al-additional-file-7-database-READ-ME.xlsx; link: https://osf.io/2h8jg)

(9) **Additional file 8:** R script used to summarise the EIPAAB Database interactive HTLM (https://jakemartinresearch.github.io/EIPAAB-database/); a static version is also available on OSF (name: martin-et-al-additional-file-8-r-script.Rmd; link: https://osf.io/2wc7f)

(10) **Additional file 9:** The ROSE flow diagram (name: martin-et-al-additional-file-9-ROSES-diagram.pdf; link: https://osf.io/pxu5y)

(11) **Additional file 10:** The Web of Science annual article counts for each of the most common research categories identified in the database (name: martin-et-al-additional-file-10-wos-research-areas-1992-2022.xlsx; link: https://osf.io/t8yja)

Supplementary Table S1: Changes to the full-text screening and data extraction form

Supplementary Table S1: Changes to the full-text screening and data extraction form after protocol publication. See Supplementary file 3 for the full data extraction form.

Question in protocol form	Brief question details	Changes made *(none, removed, changed, moved)*	Question in final form
1	Instructions (formatted as question)	**None**	1
2	Screener initials	**None**	2
3	Article ID	None	3
4	Article Title	**Removed:** This information was added after screening by cross-referencing article ID with our initial search results and DOI with search results	NA
5	DOI	**None**	4
6	Article source	**Removed:** Information contained in the DOI	NA
7	Is this article an academic thesis?	**Removed:** Academic theses were not included	NA
8	What type of thesis?	**Removed:** Academic theses were not included	NA
9	Country of the corresponding author	**Removed:** Bibliometrics analysis was not conducted. This information can be added respectively using the DOI	NA
10	Is this the first form used to extract this paper?	**None**	5
11	Inclusion criteria header	**None**	6
12	Animal class	**None**	7
13	Animal strain / genetically modified?	**None**	8
14	Is it a compound of interest?	**None**	9

15	In there a control group?	**None**	10
16	Is behaviour measured?	**None**	11
17	Publication language	**None**	12
18	Should this paper be included or excluded or discussed?	**None**	13
19	Elaborate on reasoning for exclusion	**None**	14
20	Elaborate on reasoning for discussion	**None**	15
21	How many species are used?	**Changed:** the wording of the question was changed to focus response on behavioural endpoints<br>*Original*: "How many species are studied? (maximum of 5 can be extracted with one form)"<br><br>*Revised*: "How many species of interest are used in behavioural measures? (maximum of 5 can be extracted with one form)"	16
22	What is the scientific name?	**None**	17
23	Is the study species freshwater, marine or estuarine?	**Removed:** This data would have required each screener to search for taxa-specific information for each article. Given many species appear in the database multiple times, adding this information post-screening would be more efficient	NA
24	What is the source of animals used?	**None**	18
25	Were details on the acclimation of the species to housing / experimental conditions reported?	**Removed:** This data extraction was highly inconsistent during pilot screening and was not essential to the broader focus of the map. The process and definition of acclimation were subjective and not always clear	NA
26	At what life stage was behaviour measured?	**Changed:** wording was changed to increase specificity and avoid errors:<br>*Original*: "What age class is studied for ________?"<br><br>*Revised*: "At what life stage was behaviour measured for ______? Select all that apply if behaviour is measured on multiple life stages.	19

		Tadpoles are included as larvae. Fry are included as juvenile. Larvae have emerged from the yolk sac and are therefore external from the embryo or the mother.”	
27	Is body size reported before the exposure?	**Removed:** This data was inconsistent during pilot screening and was not essential to the broader focus of the map.	NA
28	What sexes are studied?	**Changed:** wording was changed to increase specificity and avoid errors:<br>*Original*: “What sex(es) are studied for _______?”<br><br>*Revised*: “What sex(es) are used in the behavioural testing following exposure for _______?”	20
29	How many different compounds of interest were used?	**None**	21
30	What was the name of the compound?	**None**	22
31	Is the CAS reported?	**None**	23
32	Is the purity reported?	**None**	24
33	What type of exposure was conducted?	**Changed**: extra options were removed because meta-data on non-waterborne exposures was not extracted:<br>*Original*: “Q33 What type of exposure was conducted with the compound ________?<br>o Waterborne<br>o Foodborne<br>o Injection or implant<br>o Sediment”<br><br>*Revised*: “Q25 What was the primary route of exposure with the compound ________?<br>Other exposure routes include foodborne, sediment, injection or	25

		implant.<br>o Waterborne only<br>o Waterborne plus any other route<br>o Other exposure route”	
34	How many exposure groups	**None**	26
35	What was the lowest dose?	**None**	27
36	What was the units?	**None**	28
37	What was the highest dose?	**None**	29
38	What was the units of the highest dose?	**None**	30
39	Are the exposure groups on a scale of 10?	**Removed:** This data extraction was inconsistent during pilot screening and was not essential to the broader focus of the map	NA

40	Are waterborne exposure concentrations validated?	Changed to increase the specificity of the wording:<br>Original: Q40 Are the waterborne exposure concentrations for ________ measured and validated? For example, is it measured via some form of analytical chemistry or assay (e.g. LCMS, GCMS, ELISA etc.)<br>o Measured<br>o Nominal<br>Final: Q31 Are the waterborne exposure concentrations for ________ measured and validated from the behavioural experiment? For example, is it measured via some form of analytical chemistry or assay (e.g. LCMS, GCMS, ELISA etc.). They must be measured during the behavioural experiment itself. See FAQ document for further details.<br>o Measured<br>o Nominal	31
41	How many time points were behaviours measured?	**Removed**: This data extraction was highly inconsistent during pilot screening and was not essential to the broader focus of the map	NA
42	What was the minimum exposure duration before a behavioural assay?	**Changed:** the question was changed from free-form text entry to multiple choice with time brackets to avoid errors introduced by manually written answers	32
43	What was the maximum exposure duration before a behavioural assay	**Changed:** the question was changed from free-form text entry to multiple choice with time brackets to avoid errors introduced by manually written answers	33
44	Was there a depuration period?	**Removed:** this data was not essential to the broader focus of the map	NA
45	Were internal concentrations measured?	**Moved**: This question was moved to a later section of the survey because it made more sense there. See Q 48.	NA

46	Where were animals exposed?	**Changed**: less options were given to simplify choices.<br><br>*Original*:<br>o Indoor laboratory setting<br>o Assumed indoor lab setting<br>o Outdoor restricted setting (cannot interact with wild species)<br>o Outdoor natural setting<br><br>*Revised*:<br>o Indoor laboratory setting or assumed indoors<br>o Outdoor restricted setting (cannot interact with wild species)<br>o Outdoor natural setting	34
47	Behavioural section introductory text	**None**	35
48	What types of behaviours were measured?	**Removed:** This question was originally structured so that extractors would select the categories of behaviours that were in the article, and then they would select a more specific behavioural classification within that category. This question was also redundant because this information could be inferred by the choice of subcategory, so it was removed.	NA
49	Movement and activity	**None**	36
50	Courtship and mating	**None**	37
51	Post mating and parental care	**None**	38
52	Aggression	**None**	39
53	Sociality	**None**	40
54	Cognition and learning	**None**	41
55	Boldness or anxiety	**Changed**: Added one more multiple-choice option so that "novel object" was also a subcategory	42
56	Foraging	**None**	43
57	Antipredator	**None**	44

NA	NA	**Added**: A final question called "Other behaviour" was added so that if extractors had a behaviour that was not covered in one of the categories, they could add it.	45
58	Behaviour as an individual or group context?	**Change**: The options given were simplified because this question was not essential to our primary and secondary objectives<br><br>*Original*: "Q58 Behaviour in an individual or group context for exposure to _____?<br>o Individual<br>o Pair or group<br>o Both<br>o Not stated or evident"<br><br>*Final*: "Q46 Is behaviour measured in a social context following exposure to _____?<br>o Yes<br>o No "	46
59	Where was behaviour measured?	**Changed:** options were simplified<br><br>*Original*: "Q59 Where were behaviours measured after exposure to ${Q30/ChoiceTextEntryValue}? Select all that apply. Note this can be different than the exposure location.<br>▢ Indoor laboratory setting<br>▢ Assumed indoor lab setting<br>▢ Outdoor restricted setting (cannot interact with wild species)<br>▢ Outdoor natural setting"<br><br>*Revised*: "Q47 In what setting was the behaviour measured during exposure to ${Q22/ChoiceTextEntryValue}? Select all that apply. Note this can be different than the exposure location.<br>▢ Indoor laboratory setting or assumed indoors (1)<br>▢ Outdoor restricted setting (cannot interact with wild species) (3)<br>▢ Outdoor natural setting (4)"	47

NA	NA	**Moved**: Were internal concentrations measured? This question was moved here in the final extraction form.	48
60	Interactive treatments	**Removed:** This data extraction was inconsistent during pilot screening and was not essential to the broader focus of the map. Extractors' definitions of additional treatments were subjective. JMM and ESM felt that a more strict definition of what could be considered an 'interactive treatment' would depend on the structure of the statistical methods, which were not considered in this map.	NA
61	Compound mixtures	**None**	49
62	Are any sub organismal endpoints measured	**None**	50
63	Is survival growth or compounds of reproductive success measured?	**Changed**: options were simplified from multiple choice to a Boolean answer 'yes/no'	51
64	Is a guideline protocol followed?	**None**	52
65	Are validity checks for the guideline method(s) being used?	**Removed:** This data was too time-consuming to extract. It required the extractors to know what the validity checks were for guidelines. It was not essential to the broader focus of the map	NA
66	Did the study follow GLP?	**None**	53
67	Animal feeding information?	**None**	54
68	Light dark cycle description	**None**	55
69	Are water quality parameters described?	**Changed**: options were simplified from multiple choice to a Boolean answer 'yes/no'	56
70	Is the stocking density reported	**Removed** This data extraction was inconsistent during pilot screening and was not essential to the broader focus of the map. This information was not reported clearly in most articles	NA
71	Is a solvent control or sham control used	**Removed** This data extraction was inconsistent during pilot screening and was not essential to the broader focus of the map	NA
72	Were treatments randomized?	**None**	57
73	How was behaviour scored?	**None**	58

74	If scoring was automated, type the software used	**Removed:** was not essential to the broader focus of the map	NA
75	Blinded scoring?	**None**	59
76	Was commercial funding provided for the study?	**Removed**: This question was redundant, because of the following question	NA
77	Was there a conflict or competing interests?	**Changed:** this was changed to specify that this can include competing financial interests too:<br><br>*Original*: "Q77 Conflict of Interests statement. Do the authors have a conflict of interest statement? If so, do they declare a conflict?"<br><br>Revised: "Q60 Conflict or competing interests statement. Do the authors have a conflict or competing interests statement? If so, do they declare a conflict? This can be phrased as competing financial interests."	60
78	Are the data or code available?	**Removed:** This data was too time-consuming to extract. It was not essential to the broader focus of the map	NA
79	Primary motivation	**None**	61
80	Ease of extraction (rating)	**Removed:** This was deemed very subjective and likely incomparable among extractors. It was not essential to the broader focus of the map	NA
81	Elaboration and comments regarding this paper	**None**	62

Supplementary Table S2: Full search strings used in Web of Science and Scopus

Supplementary Table S2: Final search string annotations for Web of Science and Scopus. Colour coding is used to show the PECO elements, see legend box for attribution to search term categories.

**Legend**									
	**Outcome**		**Population**		**Exposure**		**Exposure environment**		**Excluded terms**

**Web of Science**
((TS = (behav* OR personalit* OR courtship* OR “parental care” OR “maternal care” OR “paternal care” OR mating OR “mate choice” OR “mate selection” OR “mate attract*” OR spawn* OR cuckold* OR nest* OR predat* OR antipredat* OR anti-predat* OR escap* OR burrow* OR cryptic OR hiding OR shelter* OR forag* OR feed* OR hunt* OR provision* OR aggress* OR schooli* OR shoal* OR social* OR affiliat* OR defen* OR contest OR dispers* OR migrat* OR swim* OR locomot* OR move* OR “activity level*” OR exploration OR anxiety OR bold* OR scototaxis OR phototaxis OR thigmotaxis OR learn* OR memory OR cognit*)) AND (TS = (“aquatic animal*” OR “aquatic wildlife” OR “aquatic organism*” OR fish OR fishs OR fishes OR teleost* OR guppy OR guppies OR poecilia OR goby OR gobies OR pomatoschistus OR trout* OR oncorhynchus OR salmo OR minnow* OR pimephales OR cyprin* OR stickleback* OR gasterosteus OR medaka OR oryzias OR danio OR gambusia OR carp* OR cyprinus OR sunfish OR lepomis OR "european sea bass" OR dicentrarchus OR bream* OR pagrus OR silverside OR menidia OR carassius OR herring OR clupea OR cod OR gadus OR killifish OR nothobranchius OR fundulus OR amphibia* OR frog* OR tadpole* OR xenopus OR rana OR turtle* OR chrysemys OR testudine* OR “aquatic insect*” OR invertebrate* OR crustacea* OR mollusc* OR snail* OR mussel* OR bivalv* OR amphipod* OR daphnia OR oyster* OR scallop* “aquatic worm*” OR “marine worm*” OR chronom* OR “marine mammal*” OR “aquatic mammal*” OR zooplankton* OR zebrafish OR mosquitofish OR killifish OR goldfish OR sunfish)) AND (TS = ("environmental estrogen" OR benzodiazepine* OR SSRI* OR SNRI OR "selective serotonin reuptake" OR "selective serotonin re-uptake" OR "drug residues" OR beta-blocker* OR “beta blocker*” OR anti-anxiety* OR antianxiety* OR psychoactive OR psychiatric OR pharmaceutical* OR medication* OR “prescription drug*” OR “illicit drug*” OR hallucinogen* OR “recreational drug*” OR antidepressant* OR anti-depressant* OR anxiolytic* OR antipsychotic* OR antimanic* OR anti-psychotic* OR anti-manic* OR anti-histamine* OR anti-convulsant* OR anticonvulsant* OR anti-epileptic* OR antiepileptic* OR antihistamine* OR analgesic* OR painkiller* OR “pain killer*” OR “pain relief” OR contracepti* OR stimulant* OR sedative* OR hypnotic* OR narcotic* OR “endocrine disrupting chemical” OR “endocrine disruptive chemical” OR “endocrine-disruptive chemical” OR “endocrine-disrupting chemical” OR “endocrine disruptor” OR edc)) AND (TS = (expos* OR tank* OR aquari* OR pool* OR treat* OR lab* OR mesocosm* OR dos* OR concentration* OR test*)) NOT (TS = (“drug discovery” OR “drug development” OR “marine corps” OR Fisher* OR “drug design” OR “essential oil”)))
**Scopus**

(TITLE-ABS(behav* OR personalit* OR courtship* OR “parental care” OR “maternal care” OR “paternal care” OR mating OR “mate choice” OR “mate selection” OR “mate attract*” OR spawn* OR cuckold* OR nest* OR predat* OR antipredat* OR anti-predat* OR escap* OR burrow* OR cryptic OR hiding OR shelter* OR forag* OR feed* OR hunt* OR provision* OR aggress* OR schooli* OR shoal* OR social* OR affiliat* OR defen* OR contest OR dispers* OR migrat* OR swim* OR locomot* OR move* OR “activity level*” OR exploration OR anxiety OR bold* OR scototaxis OR phototaxis OR thigmotaxis OR learn* OR memory OR cognit*) OR AUTHKEY(behav* OR personalit* OR courtship* OR “parental care” OR “maternal care” OR “paternal care” OR mating OR “mate choice” OR “mate selection” OR “mate attract*” OR spawn* OR cuckold* OR nest* OR predat* OR antipredat* OR anti-predat* OR escap* OR burrow* OR cryptic OR hiding OR shelter* OR forag* OR feed* OR hunt* OR provision* OR aggress* OR schooli* OR shoal* OR social* OR affiliat* OR defen* OR contest OR dispers* OR migrat* OR swim* OR locomot* OR move* OR “activity level*” OR exploration OR anxiety OR bold* OR scototaxis OR phototaxis OR thigmotaxis OR learn* OR memory OR cognit*)) AND (TITLE-ABS(“aquatic animal*” OR “aquatic wildlife” OR “aquatic organism*” OR *fish OR fishs OR fishes OR teleost* OR guppy OR guppies OR poecilia OR goby OR gobies OR pomatoschistus OR trout* OR oncorhynchus OR salmo OR minnow* OR pimephales OR cyprin* OR stickleback* OR gasterosteus OR medaka OR oryzias OR danio OR gambusia OR carp* OR cyprinus OR lepomis OR "european sea bass" OR dicentrarchus OR bream* OR pagrus OR silverside OR menidia OR carassius OR herring OR clupea OR cod OR gadus OR nothobranchius OR fundulus OR amphibia* OR *frog* OR tadpole* OR xenopus OR rana OR turtle* OR chrysemys OR testudine* OR “aquatic insect*” OR invertebrate* OR crustacea* OR mollusc* OR *snail* OR mussel* OR bivalv* OR amphipod* OR daphnia OR oyster* OR scallop* OR “aquatic worm*” OR “marine worm*” OR chronom* OR “marine mammal*” OR “aquatic mammal*” OR zooplankton*) OR AUTHKEY(“aquatic animal*” OR “aquatic wildlife” OR “aquatic organism*” OR *fish OR fishs OR fishes OR teleost* OR guppy OR guppies OR poecilia OR goby OR gobies OR pomatoschistus OR trout* OR oncorhynchus OR salmo OR minnow* OR pimephales OR cyprin* OR stickleback* OR gasterosteus OR medaka OR oryzias OR danio OR gambusia OR carp* OR cyprinus OR lepomis OR "european sea bass" OR dicentrarchus OR bream* OR pagrus OR silverside OR menidia OR carassius OR herring OR clupea OR cod OR gadus OR nothobranchius OR fundulus OR amphibia* OR *frog* OR tadpole* OR xenopus OR rana OR turtle* OR chrysemys OR testudine* OR “aquatic insect*” OR invertebrate* OR crustacea* OR mollusc* OR *snail* OR mussel* OR bivalv* OR amphipod* OR daphnia OR oyster* OR scallop* OR “aquatic worm*” OR “marine worm*” OR chronom* OR “marine mammal*” OR “aquatic mammal*” OR zooplankton*)) AND (TITLE-ABS("environmental estrogen" OR benzodiazepine* OR SSRI* OR SNRI OR "selective serotonin reuptake" OR "selective serotonin re-uptake" OR "drug residues" OR beta-blocker* OR “beta blocker*” OR anti-anxiety* OR antianxiety* OR psychoactive OR psychiatric OR pharmaceutical* OR medication* OR “prescription drug*” OR “illicit drug*” OR hallucinogen* OR “recreational drug*” OR antidepressant* OR anti-depressant* OR anxiolytic* OR antipsychotic* OR antimanic* OR anti-psychotic* OR anti-manic* OR anti-histamine* OR anti-convulsant* OR anticonvulsant* OR anti-epileptic* OR antiepileptic* OR antihistamine* OR analgesic* OR painkiller* OR “pain killer*” OR “pain relief” OR contracepti* OR stimulant* OR sedative* OR hypnotic* OR narcotic* OR “endocrine disrupting chemical” OR “endocrine disruptive chemical” OR “endocrine-disruptive chemical” OR “endocrine-disrupting chemical” OR “endocrine disruptor” OR edc) OR AUTHKEY("environmental estrogen" OR benzodiazepine* OR SSRI* OR SNRI OR "selective serotonin reuptake" OR "selective serotonin re-uptake" OR "drug residues" OR beta-blocker* OR “beta blocker*” OR anti-anxiety* OR antianxiety* OR psychoactive OR psychiatric OR pharmaceutical* OR medication* OR "prescription drug*" OR “illicit drug*” OR hallucinogen* OR “recreational drug*” OR antidepressant* OR anti-depressant* OR anxiolytic* OR antipsychotic* OR antimanic* OR anti-psychotic* OR anti-manic* OR anti-histamine* OR anti-convulsant* OR anticonvulsant* OR anti-epileptic* OR antiepileptic* OR antihistamine* OR analgesic* OR painkiller* OR “pain killer*” OR “pain relief” OR contracepti* OR stimulant* OR sedative* OR hypnotic* OR narcotic* OR “endocrine disrupting chemical” OR “endocrine disruptive chemical” OR “endocrine-disruptive chemical” OR “endocrine-disrupting chemical” OR “endocrine disruptor” OR edc)) AND (TITLE-ABS(expos* OR tank* OR aquari* OR pool* OR treat* OR lab* OR mesocosm* OR dos* OR concentration* OR test*) OR AUTHKEY(expos* OR tank* OR aquari* OR pool* OR treat* OR lab* OR mesocosm* OR dos* OR concentration* OR test*)) AND NOT (TITLE-ABS(“drug discovery” OR “drug development” OR “marine corps” OR Fisher* OR “drug design” OR “essential oil”) OR AUTHKEY(“drug discovery” OR “drug development” OR “marine corps” OR Fisher* OR “drug design” OR “essential oil”))

Supplementary Table S3: A list of CRED criteria and details

Supplementary Table S3: Criteria for Report Ecotoxicity Data (CRED) (Moermond et al. 2016, https://doi.org/10.1002/etc.3259) reliability questions and the corresponding question number in our data extraction form.

**CRED Reliability Question**	**Corresponding question in extraction form**<br><br>**(*name in the database*)**
1. Is a guideline method (e.g., OECD/ISO) or modified guideline used?	Question 52 (*validity_guideline*)
2. Is the test performed under GLP conditions?	Question 53 (*validity_good_laboratory_practice*)
3. If applicable, are validity criteria fulfilled (e.g., control survival, growth)?	Question 10, 51 (*validity_survival_growth_reproduction*)
4. Are appropriate controls performed (e.g., solvent control, negative and positive control)?	NA - requires expert knowledge on what controls would be appropriate to the study
5. Is the test substance identified with name or CAS number? Are test results reported for the appropriate compound?	Question 23 *(validity_compound_cas_reported)*
6. Is the purity of the test substance reported? Or, is the source of the test substance trustworthy?	Question 24 (*validity_compound_purity_reported*)
7. If a formulation is used or if impurities are present: Do, other ingredients in the formulation exert an effect? Is the amount of test substance in the formulation known?	NA; no formulations (i.e. only single compounds) will be included in this map.
8. Are the organisms well described (e.g., scientific name, weight, length, growth, age/life stage, strain/clone, gender if appropriate)?	Questions 7-8, 17-19 (*species_stage, species_sex*)
9. Are the test organisms from a trustworthy source and acclimatized to test conditions? Have the organisms not been pre-exposed to test compound or other unintended stressors?	Question 18 (*species_source*)
10. Is the experimental system appropriate for the test substance, taking into account its physico-chemical characteristics?	NA; not extracted, requires subjective and expert judgement
11. Is the experimental system appropriate for the test organism (e.g., choice of medium or test water, feeding, water characteristics, temperature, light/dark conditions, pH, oxygen content)? Have conditions been stable during the test?	Questions 53-56 (*validity_animal_feeding, validity_water_quality, validity_light_cycle*)
12. Were exposure concentrations below the limit of water solubility (taking the use of a solvent into account)? If a solvent is used, is the solvent within the appropriate range and is a solvent control included?	Not directly assessed, but can be calculated from Maximum dose

13. Is correct spacing between exposure concentrations applied?	NA; not extracted because what is deemed "correct" may differ between disciplines and the map will include papers from outside of ecotoxicology
14. Is the exposure duration defined?	Question 32-33 (*compound_min_duration_exposure, compound_max_duration_exposure*)
15. Are chemical analyses adequate to verify concentrations of the test substance over the duration of the study?	Question 31, Question 48 (*validity_compound_water_verification, validity_compound_animal_verification*)
16. Is the biomass loading of the organisms in the test system within the appropriate range (e.g., <1 g/L)?	NA; requires expert knowledge of the exposure system and species.
17-20. Relate to statistical design	NA, statistical design not extracted or considered in the systemic map

Supplementary Table S4: Behavioral categories and definitions

Supplementary Table S4: Behaviour categories and definition. The 'New' column indicates whether the category was added after full-text screening as a result of the 'Other' free-text entries by extractors

Name	Definition	new
**1. Movement/locomotion (6 subcategories)**		
Normal locomotor activity	Locomotor activity that is described by the authors as being under natural/normal conditions, or is not described in any specific behaviour context	0
Abnormal movement	Locomotion that is described by the authors as abnormal or atypical for the organism, (e.g. unusual circling behaviour, seizure-like behaviour)	0
Migration or dispersal	Large-scale or seasonal movement of animals from one region to another as described by the authors. Migration and dispersal were originally separate categories but were combined	0
Light-stimulated locomotor activity	Locomotor activity that is initiated or measured during/after shifting light conditions, but is not described by the authors in an anxiety/boldness context	1
Contact-stimulated locomotor activity	Locomotor activity that is initiated or measured during/after some kind of physical contact with the animal, but is not described by the authors in an anxiety/boldness or predation context	1
Auditory-stimulated locomotor activity	Locomotor activity that is initiated or measured during/after an auditory stimulus, but is not described by the authors in an anxiety/boldness or predation context	1
**2. Pre-mating and mating behaviour (7 subcategories)**		
Pre-copulatory and mating behaviour	Pre-copulatory behaviour and/or actual mating with a conspecific where they are free to interact	0
Mate choice or preference	The selection to associate with a potential mate over some other number of potential mate options	0
Nesting behaviour	Nest-building, nest residence, nest preparation, and nest acquisition behaviour in a reproductive context	0
Non-interactive pre-copulatory behaviour	Pre-copulatory behaviour that is directed at a perceived/potential mate that can not interact directly (i.e. model/video, behaviour), or pre-copulatory behaviour not directed at a specific mate (e.g. mate calling).	0

Pre-copulatory behaviour with a physical barrier	Pre-copulatory behaviour directed at a live mate, but unable to freely interact	0
Locomotor activity within this context	Locomotion measured within a reproductive context, as described by the authors	0
Direct mate competition	Direct free-interacting competition for a mate	1
**3. Post-mating behaviour (2 subcategories)**		
Offspring care	Proving resources or care for offspring (e.g. feeding, cleaning, fanning, nest tending, offspring dispersal)	0
Resource defence	Defence of a resource important for reproductive success (e.g. mate, nest, or offspring guarding/defence from a threat)	0
**4. Aggression (5 subcategories)**		
Aggression towards a mirror	Aggressive behaviour directed to a mirror image of the focal individual	0
Aggression towards a live competitor free to interact	Aggressive behaviour directed to a live competitor where they are free to interact directly	0
Aggression towards a live competitor with a physical barrier	Aggressive behaviour directed to a live competitor with no physical contact possible	0
Aggression towards a model or video	Aggressive behaviour directed to a model or video competitor	0
Locomotor activity within this context	Locomotion measured within an aggressive or competitive context, as described by the authors	0
**5. Sociality (5 subcategories)**		
Affiliation with a live conspecific behind a barrier	Interaction with a conspecific during which the focal individual cannot freely interact with the conspecific. Not described by authors as being in a reproductive context	0
Affiliation with a live conspecific free to interact	Interaction with a conspecific during which the focal individual can freely interact with the conspecific. Not described by authors as being in a reproductive context	0
Affiliation with a conspecific model or video or mirror	Interaction with a model, video, or mirror image of conspecific, such that the social pattern does not independently response to the presence of behaviour of the focal fish. Not described by authors as being in a reproductive context	0
Affiliation with a live heterospecific free to interact	Interaction with a heterospecific during which the focal individual cannot freely interact with the conspecific. Is not described by authors as being in a reproductive context	1

Undirected social behaviour	Social behaviour that is not directed at any given individual (e.g. calling). Not described by authors as being in a reproductive context	1
**6. Cognition and learning (6 subcategories)**		
Positive reinforcement associative learning	A conditioned associative behaviour with a positive stimulus (e.g. food, conspecifics, place preference)	0
Memory or information retention	Testing a learned association after time has passed	0
Negative reinforcement associative learning	A conditioned associative behaviour with a negative stimulus (avoidance leaning)	0
Habituation task	The decrease in responsiveness to a stimulus over time (desensitisation to a stimulus)	0
Novel task problem-solving	A new/novel challenge or context that has a solution criterion (e.g. maze or puzzle box)	0
Locomotor activity within this context	Locomotion measured within a learning or cognition context, as described by the authors	0
**7. Anxiety and boldness (9 subcategories)**		
Anxiety response to light	Anxiety-like responses measured during changes in lighting conditions (e.g. lighting on-off, lighting intensity changes), as described by the authors	0
Anxiety response in a novel tank or open-field or exploration assay	Anxiety-like responses measured during a novel tank (novel environment), open-field, or exploration assay, as described by the authors	0
Scototaxis-related anxiety response	Anxiety-like responses measured using a scototaxis assay, through the preference for a dark or light area (black/white), as described by the authors	0
Sheltering or hiding	Emergence from, or preference to stay in, a shelter or hiding area (of perceived safety), as described by the authors. Not described in a predation context.	0
Shoaling tendency	Anxiety-like responses measured as the tendency of an animal to form a social group or to move toward conspecifics	0
Locomotor activity within this context	Locomotion measured within an anxiety context, as described by the authors	0
Novel object	Anxiety-like responses measured as the tendency of an animal to approach or associate with a novel object	0
Stress related response to simulated attack	Anxiety-like response to a simulated attack (e.g. chasing with a net, or prodding the animal), without mention of a predator context, as described by the authors	1

Stress related feeding	Anxiety-like response measured during feeding (e.g. time taken to eat a food item), as described by the authors	1
**8. Foraging and feeding (5 subcategories)**		
Feeding on a motile food source (live)	Feeding or foraging behaviour on a food source that is live and/or motile	0
Feeding on a non-motile food source (not live)	Feeding or foraging behaviour on a food source that is not live and/or not motile, includes filtration behaviour, foraging on algae	0
Locomotor activity within this context	Locomotion measured within a foraging/feeding context, as described by the authors	0
Feeding on unspecified food source	Feeding or foraging behaviour on a food source that is not specified	1
Responsiveness to food cue	Response to the presence of food cues without direct access to the food source (e.g. chemical cues)	1
**9. Antipredator behaviour (5 subcategories)**		
Response to a simulated predator	The response of the focal fish to the presence of, or attack by, a simulated predator (e.g. marble drop or model or chasing), as described by the authors	0
Response to a predation olfactory cue	The response of the focal fish to predation olfactory cues (e.g. predator and conspecific alarm cues), as described by the authors	0
Response to a live predator behind a barrier	The response to a live predator that cannot directly access the focal individual, as described by the authors	0
Response to a live predator free to interact	The response to a live predator that can directly access the focal individual, as described by the authors	0
Locomotor activity within this context	Locomotion measured within a predator context, as described by the authors	0
**10. Other behaviours not categorised (12 subcategories)**		
Attraction or aversion to compound	Measures the attraction or aversion of the animal to the test compound	1
Pain response	A pain-induced behavioural response	1
Colour preference	An innate or unconditioned preference for colour	1
Camouflage	The camouflage (colour change) behaviour of an animal. Not necessary in an antipredator or stress-related response	1
Sedation	Sedation-like effects of the test compound	1
Place preference	An innate or unconditioned preference for a given area	1

Home construction	The construction of a burrow or dwelling. Not necessary as an antipredator or stress-related response, or in a reproduction context	1
Righting behaviour	The ability of the animal to return to proper orientation	1
Exercise challenge	A physical challenge such as a critical swimming speed	1
Circadian activity	Measurements of movements over wake and rest cycles (e.g. day and night)	1
Electric discharge	The discharge of electric currents by the animal	1
Lateralization	The tendency for behavioural task to be approached or taken using the left or right side of the body	1

Supplementary Table S5: Behavioral scoring methods

Supplementary Table S5: Method(s) used to score behaviours and definitions for each. The 'New' column indicates whether the category was added after full-text screening as a result of the 'Other" free-text entries by extractors.

Name	Definition	New
Supervised automated tracking approaches	Approaches used to track or quantify animal behaviour from a video that is automated, but supervised by a human user (e.g. EthoVison, IDtackerAI)	0
Manual or human scoring from videos or image	Manual scoring by a human observer, which is done from a video; this can be done completely manually or with an ethogram/key-logging software (e.g. BORIS, Jwatcher).	0
Live scoring in real-time	Manual scoring by a human observer in real-time (not from video)	0
Not specified	The scoring of behaviour was not specified	0
Quantifying food consumption	Feed or foraging was scored indirectly by quantifying the amount of food items consumed	1
Acoustic telemetry	Behaviour scored using acoustic telemetry techniques	1
Sensor for physical movement	e.g. optical mechanical sensor, vibration sensory, electrical activity to track movement	1
Acoustic analysis software	Software used to quantify vocalisations (e.g., mating calls)	1
Other	Anything that does not fit into the above categories.	1

Supplementary Table S6: Metadata consistency estimates

Supplementary Table S6: Consistency estimates for each metadata category, estimated by comparing the metadata extracted for all articles that were screened in duplicate (*n* = 84 articles, with a total of 143 unique species by compound combinations). For each article-compound-species combination, the consistency of metadata was scored as a Boolean value (i.e. 1 or 0). Only when the metadata matched exactly were the two extractions considered consistent (e.g. even where there were multiple-choice answers, all had to match). The average consistency for each metadata were then calculated and are present below.

Metadata	Consistency
animal_class	1.0000
biomarkers	0.8812
compond_mixture	0.9021
compound_behav_agression	0.9721
compound_behav_agression_boolean	0.9861
compound_behav_antipredator	0.9651
compound_behav_antipredator_boolean	0.9861
compound_behav_boldness	0.676
compound_behav_boldness_boolean	0.9441
compound_behav_cognition	0.9791
compound_behav_cognition_boolean	0.9861
compound_behav_foraging	0.9511
compound_behav_foraging_boolean	0.9861
compound_behav_is_social	0.9301
compound_behav_location	1.0000
compound_behav_mating	0.9161
compound_behav_mating_boolean	0.9861
compound_behav_movement	0.7063
compound_behav_movement_boolean	0.7553
compound_behav_noncat	0.9931
compound_behav_noncat_boolean	0.9931
compound_behav_post_mating	0.9651
compound_behav_post_mating_boolean	0.9721
compound_behav_sociality	0.8812
compound_behav_sociality_boolean	0.9301
compound_cas	1.0000
compound_expose_route	0.9861
compound_groups	0.9371
compound_internal_conc	0.9441
compound_location	0.9931
compound_max_duration	0.8882

compound_max_std	0.9138
compound_measured	0.9721
compound_min_duration	0.8602
compound_min_std	0.9418
compound_n	0.8952
compound_purity	0.9791
motivation	0.9091
quality_animal_feeding	0.9441
quality_behav_blinding	0.9791
quality_behav_scoring	0.6084
quality_conflict	0.9931
quality_glp	0.9931
quality_guideline	0.9651
quality_light_cycle	0.9581
quality_randomization	0.8392
quality_survival_growth_reproduction	0.8392
quality_water	0.9628
species_n	0.9931
species_sex	0.9371
species_source	0.8322
species_stage	0.8252

Supplementary Table S7: Species metadata summary

Supplementary Table S7: This table shows the habitat, life stage, sex, and source metadata for species in the EIPAAB database. All values

Species metadata	Environmental	Medical	Basic research
**Habitat** (*INCU data available for 82.6%)*			
Freshwater	73.8%	94.5%	80.9%
Marine	26.2%	5.5%	19.1%
**Life stage** (*stage reported for 83.0% of species*)			
Adult	48.6%	60.1%	59.8%
Juvenile	18.9%	6.3%	13.9%
Larvae	26.1%	28.7%	23.0%
Egg or embryo	6.4%	4.9%	3.3%
**Sex** (*sex reported for 53.0% of species*)			
Female	43.0%	47.9%	45.3%
Male	57.0%	52.1%	54.7%
**Source** (*source reported for 84% of species*)			
Wild collected	35.5%	4.8%	13.1%
Lab stock from wild population	5.3%	0.5%	5.1%
Lab stock of undisclosed origin	25.0%	33.3%	22.6%
Lab stock from commercial supplier	6.1%	8.5%	7.3%
Commercial supplier or fish farm	28.2%	52.9%	51.8%

Supplementary Figure S1: Compound decision tree

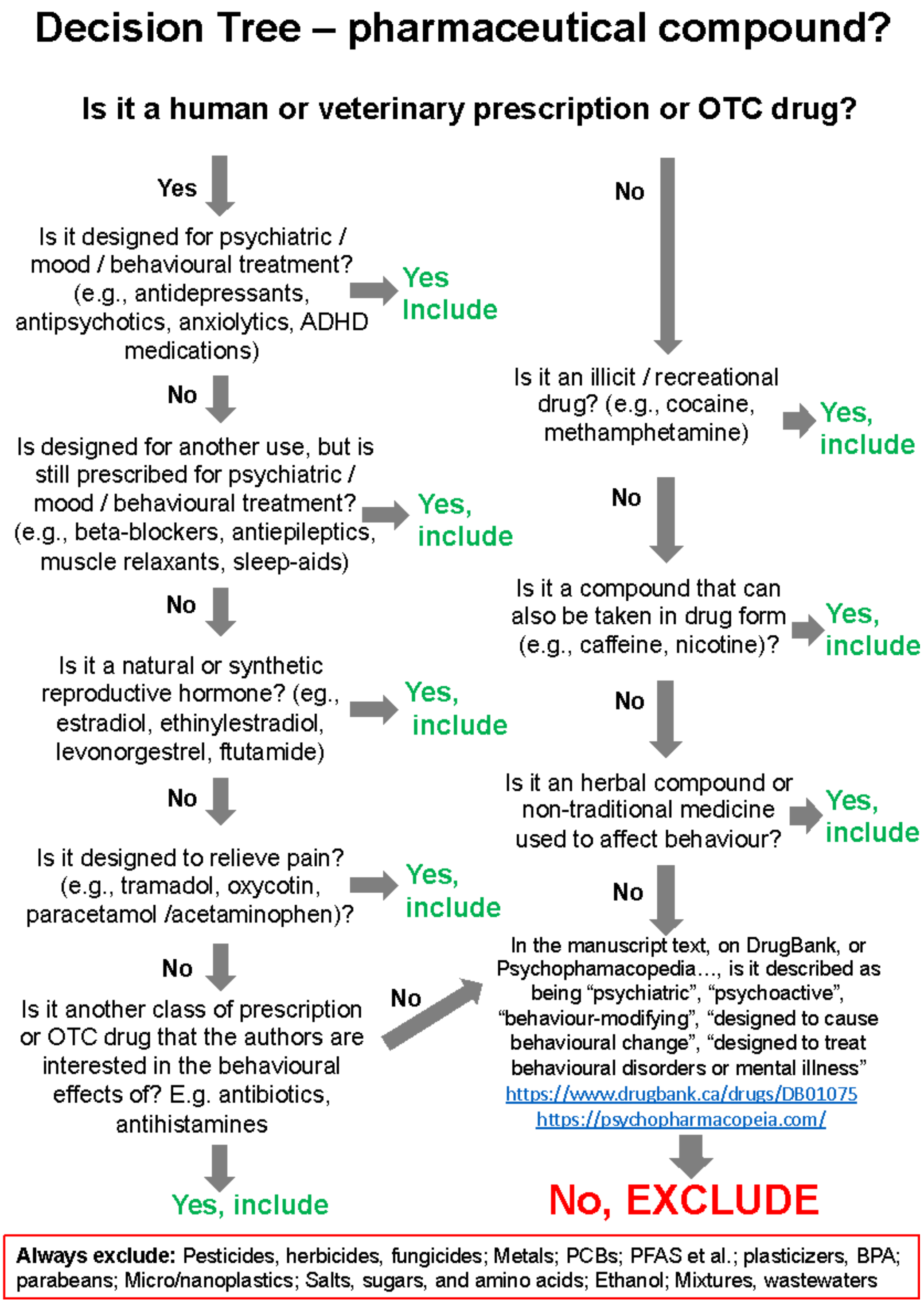

Supplementary Figure S1: Decision tree to assist extractors in deciding if the pharmaceutical compound should be included in the systematic map. OTC means "over the counter".

Supplementary Figure S2: Distribution of species use

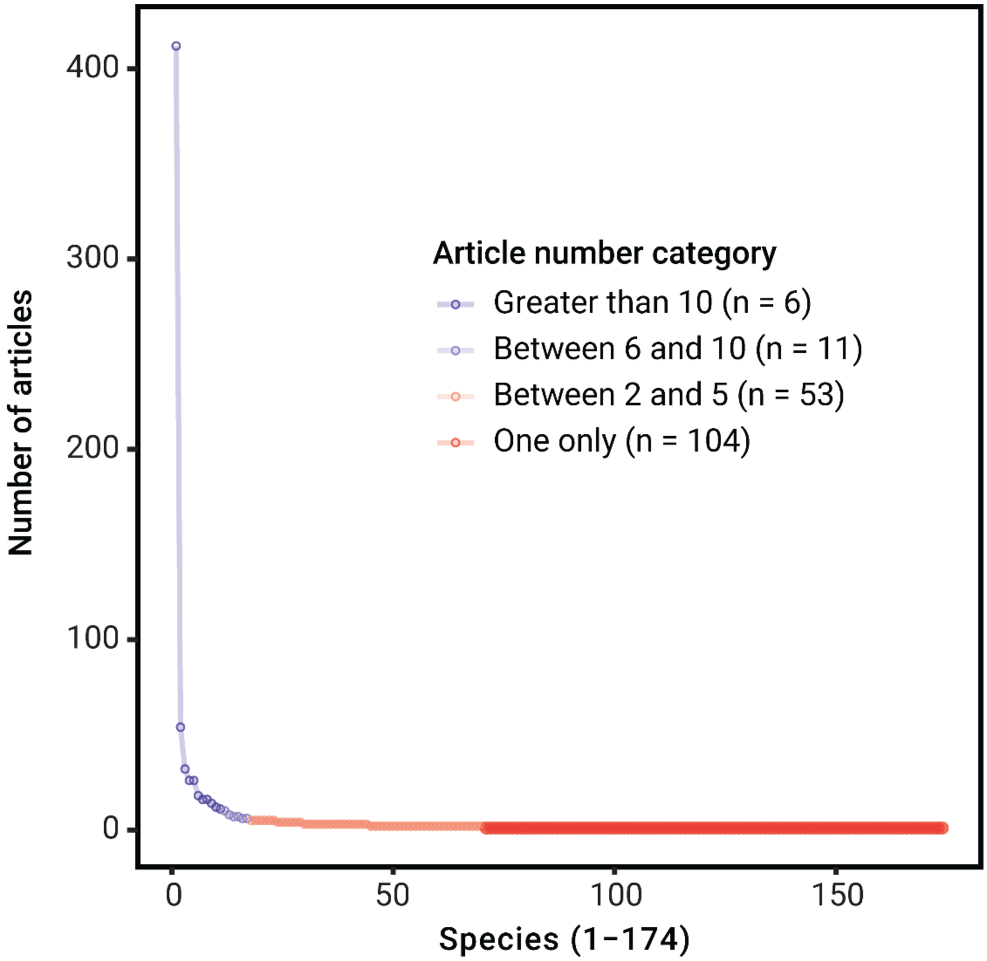

Figure S2. Overall distribution of species used in the database. The x-axis shows each species (1 to 174) arranged by the total number of studies for which it occurs. The colours are used to group species into broad use categories.

Supplementary Figure S3: Taxonomic use by study motivation

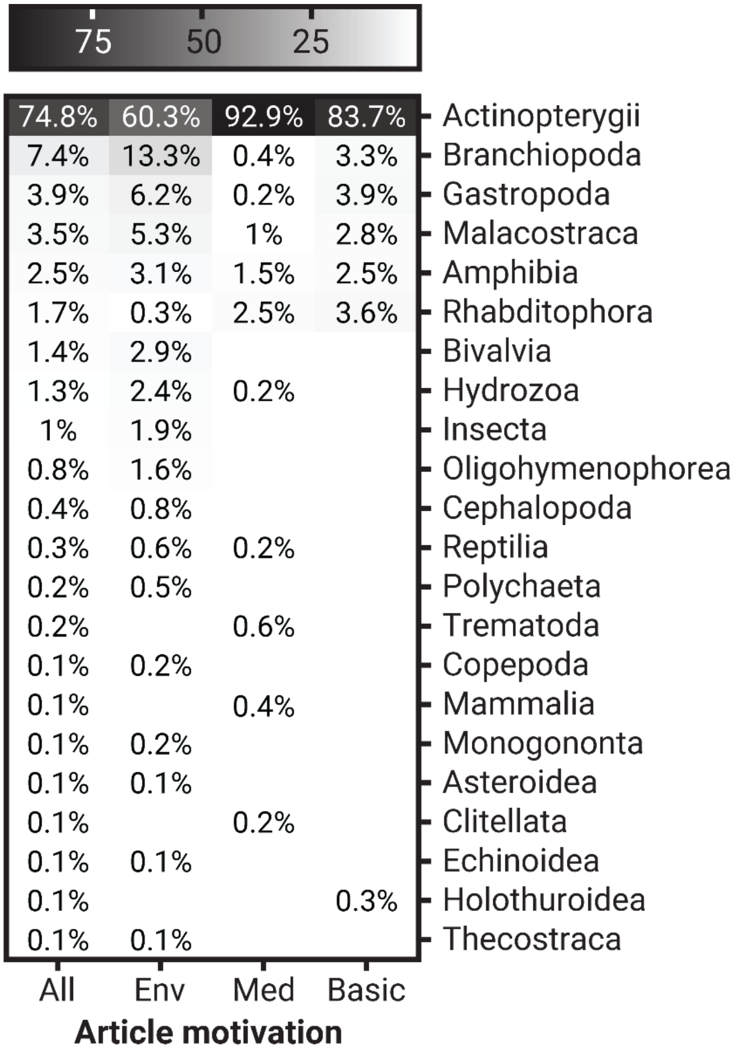

Figure S3. Taxonomic Class use for all evidence in the EIPAAB database (all), that with an environmental focus (Env), a medical focus (Med) and a Basic research focus (Basic). The columns are arranged by overall contribution to the database (i.e. the ‘All’ column). The parentage use is calculated within each study's motivation. The colour of the cell represents the relative use, with darker cells being more abundant.

Supplementary Figure S4: Number of compounds used per study

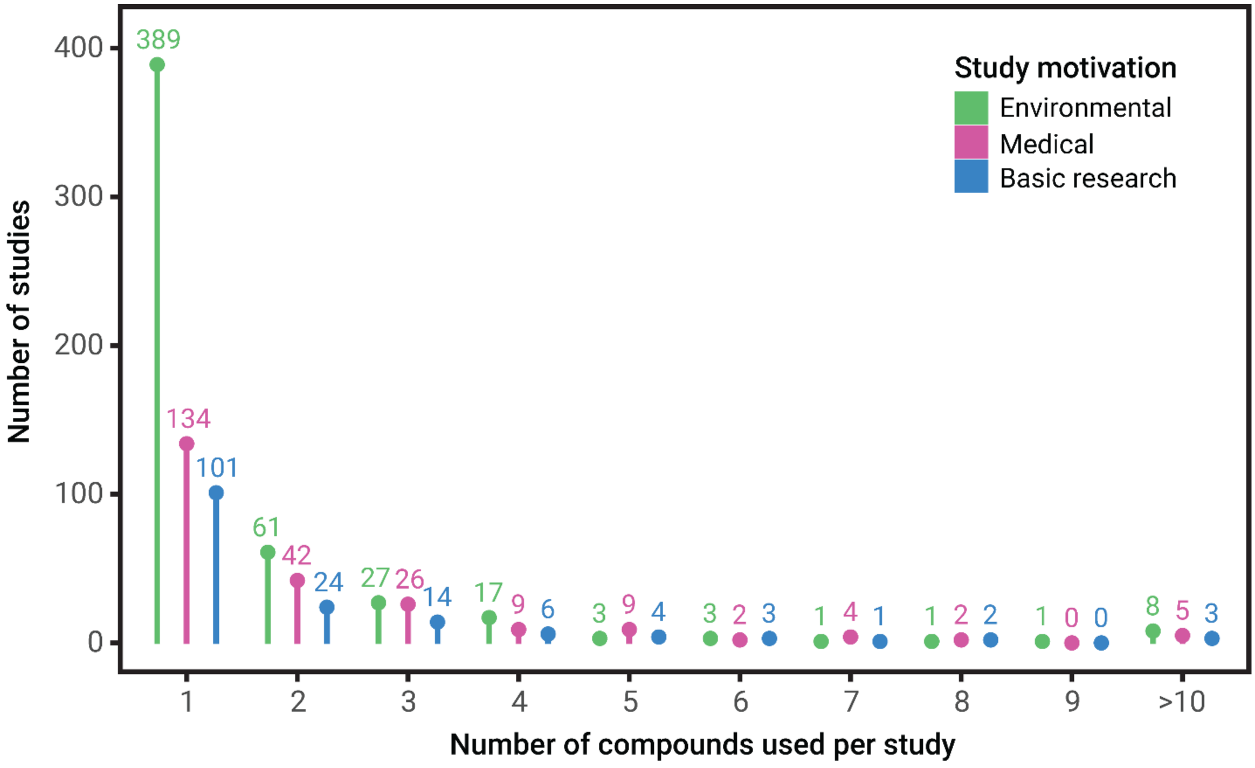

Figure S4. The number of compounds used per study, shown by study motivation.

Supplementary Figure S5: Therapeutic class level one

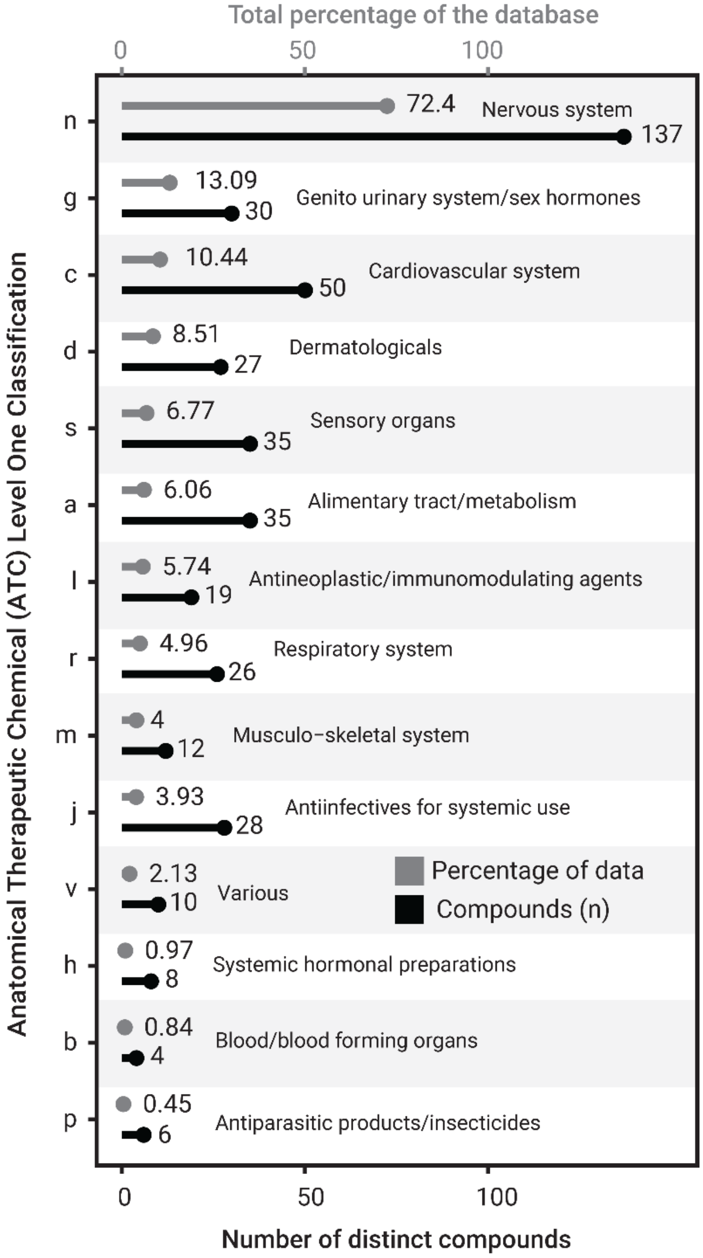

Figure S5. The fourteen level three ATC pharmacological groups, as shown by the percentage of overall occurrence in the database (grey), in addition to the number of distinct compounds within each group (black).

Supplementary Figure S6: Behavioural sub-categories

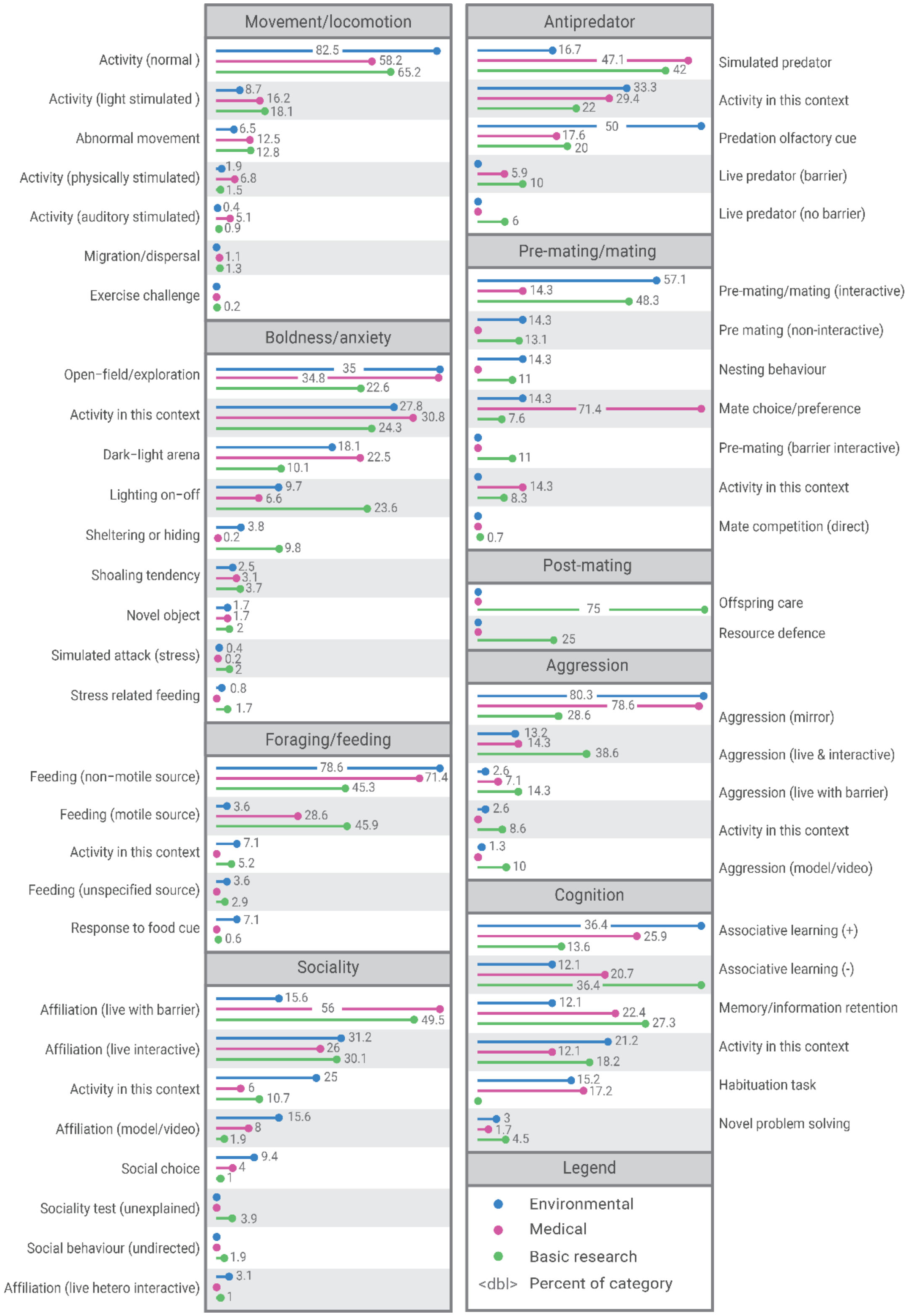

Figure S6. The percentage of each parent behavioural category that each sub-category contributes is shown separately for each study motivation.

Supplementary Figure S7: Example links between population, exposure, outcome

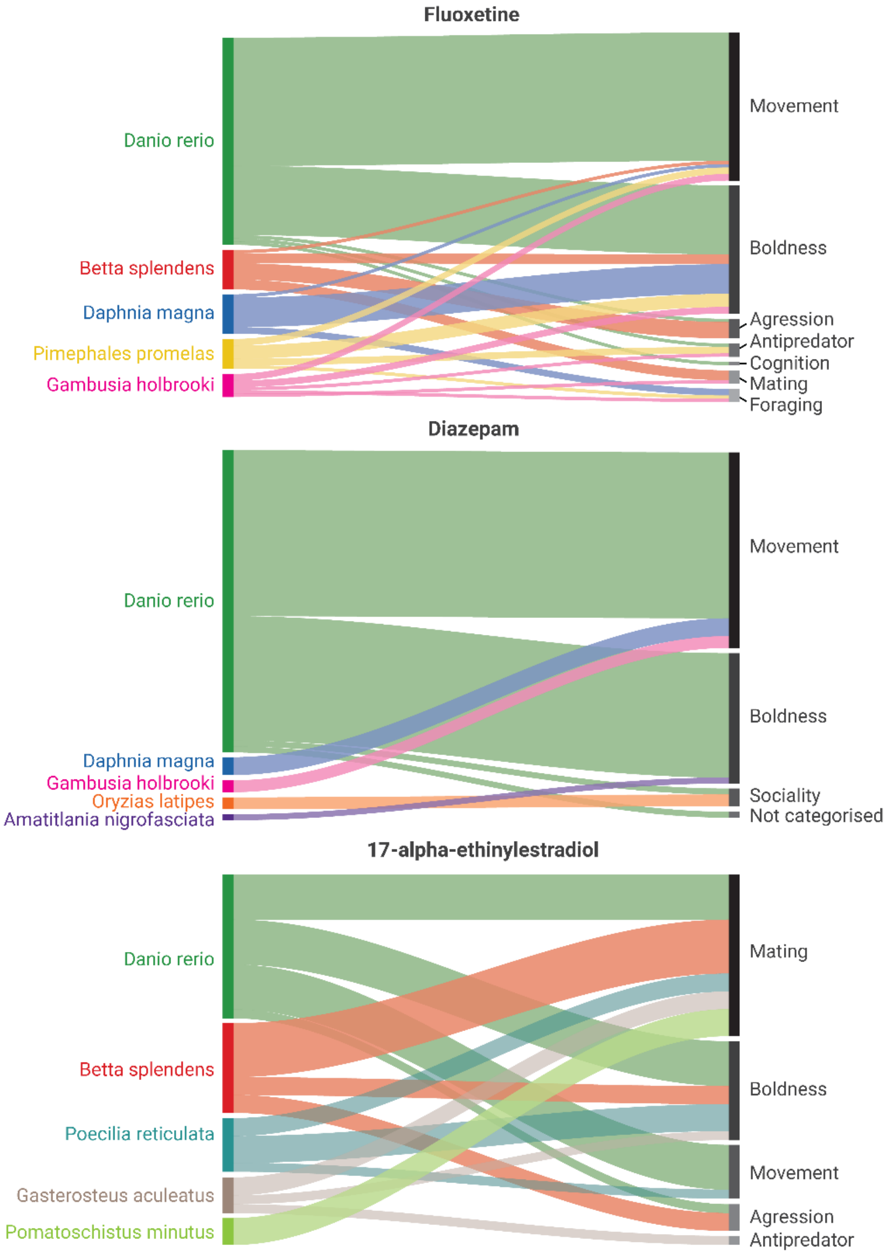

Figure S7: Example links between the population, exposure, and outcome elements using Fluoxetine, Diazepam, and 17-alpha-ethinylestradiol (the three most common compounds) as examples. The thickness of each band corresponds to the number of occurrences in the EIPAAB database.
